# Combined Effects of Pesticides and Electromagnetic-Fields on Honeybees: Multi-Stress Exposure

**DOI:** 10.3390/insects12080716

**Published:** 2021-08-10

**Authors:** Daniela Lupi, Marco Palamara Mesiano, Agnese Adani, Roberto Benocci, Roberto Giacchini, Paolo Parenti, Giovanni Zambon, Antonio Lavazza, Maria Beatrice Boniotti, Stefano Bassi, Mario Colombo, Paolo Tremolada

**Affiliations:** 1 Department of Food, Environment and Nutritional Sciences (DEFENS), University of Milan, 20133 Milan, Italy; palamaramesiano.marco@gmail.com (M.P.M.); mario.colombo@unimi.it (M.C.); 2 Department of Environmental Science and Policy, University of Milan, 20133 Milan, Italy; agnese.adani@gmail.com (A.A.); paolo.tremolada@unimi.it (P.T.); 3 Department of Earth and Environmental Sciences, University of Milano-Bicocca, 20126 Milan, Italy; roberto.benocci@unimib.it (R.B.); roberto.giacchini@unimib.it (R.G.); Paolo.parenti@unimib.it (P.P.); giovanni.zambon@unimib.it (G.Z.); 4 Istituto Zooprofilattico Sperimentale della Lombardia ed Emilia Romagna “Bruno Ubertini”, 25124 Brescia, Italy; antonio.lavazza@izsler.it (A.L.); mariabeatrice.boniotti@izsler.it (M.B.B.); stefanobassi1952@libero.it (S.B.)

**Keywords:** multi-stress approach, honeybees, biomarkers, pesticides, electromagnetic fields, stress effects

## Abstract

**Simple Summary:**

Multi-stress conditions are considered the most putative cause of honeybee decline. The ongoing reduction of domestic and natural pollinators is considered a very severe signal of the current loss of biodiversity, and it requires a broad research effort to clarify the causes. In this research, the combined effects of two possible stress sources for bees, pesticides and electromagnetic fields (multi-stress conditions) were analyzed by a field trial. After one year of monitoring, a complex picture of several induced effects was present, especially in the multi-stress site, such as disease appearance (American foulbrood), higher mortality in the underbaskets (common to pesticide-stress site), behavioral alterations (queen changes, excess of both drone-brood deposition and honey storage) and biochemical anomalies (higher ALP activity at the end of the season). The multi-stress site showed the worst health condition of the bee colonies, with only one alive at the end of the experimentation out of the four ones present at the beginning.

**Abstract:**

Honeybee and general pollinator decline is extensively reported in many countries, adding new concern to the general biodiversity loss. Many studies were addressed to assess the causes of pollinator decline, concluding that in most cases multi-stress effects were the most probable ones. In this research, the combined effects of two possible stress sources for bees, pesticides and electromagnetic fields (multi-stress conditions), were analyzed in the field. Three experimental sites were chosen: a control one far from direct anthropogenic stress sources, a pesticide-stress site and multi-stress one, adding to the same exposure to pesticides the presence of an electromagnetic field, coming from a high-voltage electric line. Experimental apiaries were monitored weekly for one year (from April 2017 to April 2018) by means of colony survival, queen activity, storage and brood amount, parasites and pathogens, and several biomarkers in young workers and pupae. Both exposure and effect biomarkers were analysed: among the first, acetylcholinesterase (AChE), catalase (CAT), glutathione S-transferase (GST) and alkaline phosphatase (ALP) and Reactive Oxygen Species (ROS); and among the last, DNA fragmentation (DNAFRAGM) and lipid peroxidation (LPO). Results showed that bee health conditions were the worst in the multi-stress site with only one colony alive out of the four ones present at the beginning. In this site, a complex picture of adverse effects was observed, such as disease appearance (American foulbrood), higher mortality in the underbaskets (common to pesticide-stress site), behavioral alterations (queen changes, excess of honey storage) and biochemical anomalies (higher ALP activity at the end of the season). The overall results clearly indicate that the multi-stress conditions were able to induce biochemical, physiological and behavioral alterations which severely threatened bee colony survival.

## 1. Introduction

The hive is a complex system regulated by many biological, chemical and nutritional factors; all the specimens contribute to the family maintenance by a highly coordinated activity. This equilibrium is daily at risk as workers patrol an environment highly modified and full of anthropogenic pollutants, especially in more developed countries. Therefore, poor nutrition and starvation [1,2,3], diseases [4,5], mites [6,7], habitat losses and fragmentation [1,8], contamination by pesticides and other pollutants [9], electromagnetic fields [10,11,12] and general environmental stresses including climate change [6,13] have been indicated as possible causes of the general bee decline and of the phenomenon called colony collapse disorder (CCD) [14,15,16,17,18].

Currently, multi-etiological factors are recognized as the most probable cause of CCD occurring in different parts of the world [19,20,21,22,23]. Stressed bees are more prone to develop disease and are able to resist less to external pressures, setting up a cascade effect [24,25,26]. When honeybees are exposed simultaneously to multiple chemicals they may exhibit, as a consequence, synergistic, additive or antagonistic effects [11,27,28]; pesticides may have a fundamental role in compromising colony health and increasing the susceptibility of the colony to pests and pathogens [7,29,30].

Sub-lethal and long-term effects can act together giving unpredictable consequences, since chronic exposure to adverse stressors gradually weakens colonies, decreases their performance, progressively reduces queen fecundity and modifies the ability of workers to learn and forage [31,32,33]

However, while many are the studies reporting the effects of a single stress factor [34,35,36,37], fewer and more recent are the studies evaluating the role of multiple-factor interaction on honeybees [38,39,40,41]. Studies of combined stressors in the laboratory allow for adding information on direct effects on bees but are far from approaching the complexity of the real world. Thus, field studies are essential for understanding combined stress effects on honeybees, even if their complexity in terms of environmental variables and interfering factors makes the interpretation of the results often difficult. A previous work [11] considered the effects of pesticides and electromagnetic fields, but separately in different experimental sites (single stress conditions). That study revealed that electromagnetic stress induced a wide enzymatic over-activation at the end of the season. According to other literature findings [42,43], this enzymatic over-activation was related to a behavioral over-activation in a period in which bees should prepare themselves to over-wintering, posing potential problems to winter survival [11]. 

The present research proposes a multi-stress approach for studying the combined effect of pesticides and electromagnetic fields with an in-field experimental trial. Experimental apiaries were exposed to pesticide stress and to multi-stress conditions (pesticides and electromagnetic field). The electromagnetic field source was determined by a high-voltage transmission power line passing just above the multi-stress location, while exposure to pesticides was common to multi-stress locations because both were positioned within an orchard farm where many chemical treatments with insecticides and fungicides were performed nearly continuously from March to October. The two stress sites, about 300 m away, were near enough to be exposed by foraging activities to the same exposure to pesticides, but sufficiently distant to be differently exposed to the electromagnetic fields caused by the high-voltage transmission line. Experimental hives in the exposure sites were compared to those in a control one located 15 km away in a natural vegetation context. Control and exposure hives were checked weekly for population parameters and health status of the colonies. A battery of biomarkers was used to detect biochemical variations and cellular stress parameters. Among the first, the quantification of Reactive Oxygen Species (ROS) and four different enzymatic activities were tested concerning neurotransmission function (acetylcholinesterase, AChE), antioxidation response (catalase, CAT), detoxification pathways (glutathione S-transferase, GST) and metabolic efficiency (alkaline phosphatase, ALP). Among cellular stress parameters, damages at the genetic level were analyzed by the quantification of the DNA fragmentation (DNAFRAGM) and those to membrane functionality by the quantification of the lipid peroxidation level (LPO).

Specific aims of this research include: (i) biomarker analysis as an early warning tool for the identification of stress factors; (ii) analysis of stress induced by exposure to pesticides; (iii) evaluation of the effects induced by multi-stress conditions (pesticides and electromagnetic field).

## 2. Materials and Methods

### 2.1. Experimental Sites

**Figure 1:**
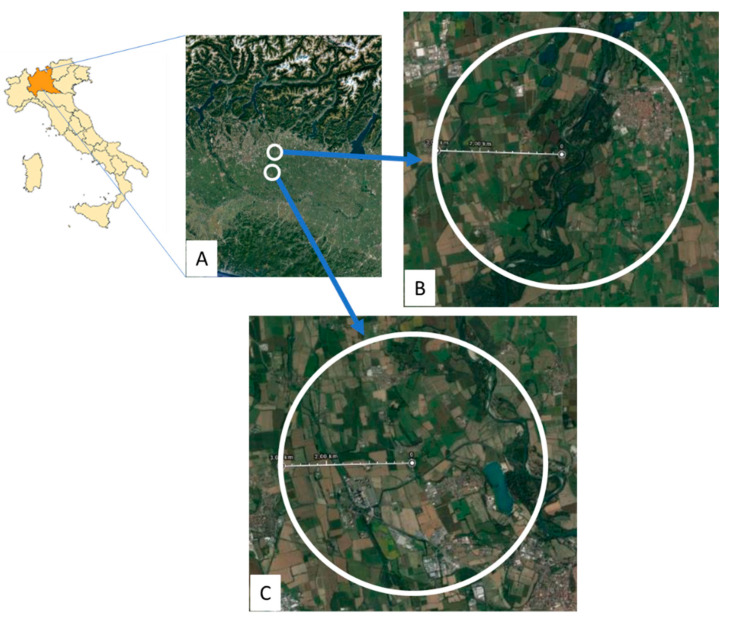
Satellite view of the experimental sites with a radius of 3 km around them; North Italy and Lombardy Region (**A**), control site at Corneliano Bertario (**B**) and exposure sites at Arcagna, centered in the same area at this scale (**C**) (https://www.google.it/maps visioned on 28 January 2021).

Experimental sites’ location and land use are shown in Figure 1. Control site was located at Corneliano Bertario (Milan Province) (45°46′14.16″ N of latitude and 9°48′75.65″ E of longitude) in an almost natural area, mainly characterized by natural vegetation, extensive agriculture and small human settlements, while the exposure sites were located at Arcagna (Lodi province), 15 km in the South (45°20′18.20″ N of latitude and 9°27′5.07″ E of longitude), inside an intensive agricultural area. Here, two hive locations (pesticide and multi-stress) were set up inside an orchard farm of the University of Milan, where a cultivar collection of different fruit species is maintained for experimental, teaching and productive purposes. The same farm is crossed, on one hedge, by a high-voltage transmission power line; hives of the multi-stress location were located just below the line and thus exposed to an electromagnetic field.

In each experimental site (control, pesticide- and multi-stress), four colonies of *Apis mellifera* were set up in spring 6 April 2017 (Figure S1). All colonies came from the same beekeeper and were randomly distributed across the experimental sites. They were selected just before the installation as they were homogeneous in terms of strength with workers distributed on ten combs and with a young and productive queen of the same age (the year before) and with the same origin (breeding lines carrying *Apis mellifera ligustica* genetic background), and they were all healthy as no disease of relief was signaled by previous owner or reported on veterinary certificate. During the trial, they were checked weekly for population parameters and health status and they were sampled monthly for biomarker analyses. Population surveys and biomarker sampling were performed from spring 2017 until spring 2018, generally the same day in all sites; only occasionally, for practical reasons, was the survey and the sampling in one site shifted the day after. To control varroa mites, on 7 July, queens in all colonies were caged to stop oviposition and oxalic acid treatment was performed after 24 days with Api-Bioxal^®^ solution (Chemicals Laif SpA, Padua, Italy) following the label doses (5 mL per bee space) [44].

### 2.2. Population Parameters and Health Status of the Colonies

Every week, when meteorological conditions were favorable, from April 2017 until November 2017 and in April 2018, a full inspection of experimental hives was executed to determine the health status of the colony, and, monthly, a sampling was performed for biomarker and virus analyses. The analyzed parameters are summarized in Table 1.

#### 2.2.1. Comb Inspection and Analysis

At each visit all the combs in the hives were singularly inspected for the visual detection of the queen; capped and uncapped brood, drone brood, honey/nectar and beebread were calculated by visual estimation on the whole family as the sum of portion in each comb, as in Liebefeld’s Method [45]. 

In addition, one comb among the central ones with the contemporary presence of capped and uncapped brood was used to estimate the size of brood area and reservoirs with an image-based. After the choice and the check of the absence of the queen on the comb, most adult bees were removed by quickly shaking the combs over the hive. A picture of both sides of the comb was taken using a Canon EOS DS126181 Camera equipped with a Canon EF-S 18-55-mm Macro lens (Canon Inc., Tokyo, Japan) and the photographs were subsequently analyzed with the program Image J [a Java-based image processing program, Laboratory for Optical and Computational Instrumentation (LOCI), UW–Madison MD, USA], able to calculate area of user-defined selections (Figure S2). Area of worker brood, drone brood, honey and pollen (in cm^2^) were obtained for each side of the comb. Taking into account that the variability of the occupied area between the two sides of the comb was limited (mean % variability coefficient of 13.8, 23.9, 19.6 and 37.1 for worker brood, drone brood, honey and pollen, respectively), the mean area of the two sides of the comb was considered to obtain information on the colony development over time.

#### 2.2.2. Mortality in Underbaskets

As the number of dead bees in front of the hive is strictly related to the health status of the colony, hives were equipped with collecting traps (underbaskets) according to [46]. Each underbasket (dimensions: base 100 cm × 60 cm; height 11 cm) was made of wood, with a fine mesh plastic net at the base and a net with a grid of 1.5 cm at the top, allowing the collection of dead bees, but avoiding predation from birds or mammals (Figure S3). Underbaskets were inspected at each visit, and all the content removed and brought to the laboratory, where dead worker bees, drones, queens, pupae and larvae were selectively counted. In each caste, specimens were inspected to check for wing deformity or other visible morphological abnormalities in workers and drones as in [56,57].

#### 2.2.3. Varroa Mite Monitoring 

To check for varroa mite infestation, adhesive sticky boards were positioned under the grid on the drawer at the bottom of the hives and removed and changed at each visit to check for naturally fallen varroa mites (Figure S4A). Adult mites captured by each board were then counted in laboratory.

In addition, powdered sugar (150 g per hive) was sprinkled on bees over the top bars of the frames and brushed between the tops of the frames with a bee brush to stimulate grooming behaviour and remove mites from bees (Figure S4B). After 1 h the sugar containing fallen varroa mites was collected from the drawer at the bottom of the hive for subsequent counting. The procedure was performed the 18 May, the 21 June, the 13 October 2017 and the 18 April 2018.

#### 2.2.4. Virus Monitoring

Acute bee paralysis virus (ABPV) and deformed wing virus (DWV) were analysed in dead workers collected from underbaskets. ABPV and DWV were considered as it is known that these two viruses are strictly linked to the depopulation syndrome induced by a series of causes, the main but not the only one of which is certainly varroa infestation. Furthermore, the presence of stressful factors of various kinds on colonies infested in a latent state with these viruses can lead to an exacerbation of the clinical form, till the point of colony collapse [58,59]. In addition, these two viruses have long been known to occur and spread in Italian colonies where they have been reported repeatedly over the last 30 years [60,61,62]. Combining these aspects, it was therefore decided to investigate and quantify the persistence of DWV and ABPV.

Full methodology is available in Supplementary Materials of the online version of this article. Briefly, bees were homogenized in MEM, and the homogenate clarified by a centrifugation. Total RNAs were purified from the supernatant using NucleoMag Virus VET Kit (Macherey-Nagel, Düren, Germany) for ABPV and One-For-All Vet Kit (Qiagen, Hilden, Germany) with KingFisher Flex automated extraction system (Thermo Fisher Scientifi Waltham, Massachusetts, USA) for DWV. Complementary DNA synthesis and amplification reaction were performed using QuantiTect Probe RT-PCR Kit (Qiagen, Hilden, Germany) in a one-step Real-Time PCR. Results were expressed in viral genome copies per bee on a basis of a calibration curve. 

#### 2.2.5. American and European Foulbrood Monitoring 

The surveillance and the monitoring of American (causative agent *Paenibacillus larvae*) and European foulbrood (causative main agent *Melissococcus plutonius*) was based mainly on clinical observation of the combs of the apiaries.

To avoid the spread of potential diseases, very strict measures were adopted since the beginning of the experimentation. Each site was provided of dedicated instrument for the inspection of the colonies and disposable gloves were used during the inspection and changed moving from one colony to the other even inside the same site.

The presence of the spores of *P. larvae* was estimated on detritus collected from the sticky boards used for *Varroa* monitoring and analyzed with a cultural method. Full methodology is available in Supplementary Materials of the online version of this article. Briefly, 1 g of debris or powdered sugar was added to sterile distilled water and heated at 85 °C. After the heat treatment, samples were plated onto culture medium and, after incubation, colonies with a *P. larvae*-like morphology were tested for catalase reaction and the catalase-negative colonies were subjected to Gram staining for confirmation. The number of viable spores was calculated and expressed as Colony Forming Units (CFU) per gram (debris or powdered sugar). The limit of detection (LOD) of the methods was 20 CFU/g.

Methods for laboratory detection of *M. plutonius*, which indeed were not detected throughout the whole experiment, were available and could have been used in case of need. They included both cultural methods (Agar Bailey medium) and PCR techniques [63,64].

#### 2.2.6. Biomarker Sampling and Analyses

Biomarker sampling was performed on nine dates (the 12 April, the 10 and the 24 May, 15 June, 3 July and 27 July, 14 September, 14 October 2017 and 17 April 2018). From each colony and each sampling data, eight worker bees and eight pupae (milk-white eye) were randomly collected and, after closing each one in a single Eppendorf marked for hive, treatment and date, they were immediately frozen in liquid nitrogen. Young-worker bees as well as pupae were exposed continuously to the electromagnetic field in the multi-stress location, as this bee caste, differently from forager bees, stays always within the hive. Pupae were chosen instead of larvae as they are supposed to integrate exposure to pesticides for all the larval stages. In each location at every sampling date, 32 pupae and 32 young workers were sampled. Pupae were collected by a pair of tweezers from a brood comb where pupal stage was present, while young workers were collected among those which did not fly back to the hive after having shaken in a box with a brood comb with bees on it. Pupae and bees were put in single vials, immediately frozen in liquid nitrogen, and carried to the laboratory for biomarker analyses. A battery of seven biomarkers was measured on each single bee. The battery was composed of four enzymatic activities: acetylcholinesterase (AChE), catalase (CAT), glutathione S-transferase (GST) and alkaline phosphatase (ALP), and three non-enzymatic biomarkers: reactive oxygen species (ROS), DNA fragmentation (DNA_FRAGM_) and lipid peroxidation (LPO). Two set of samples were taken each time: one for measuring the four enzymatic activities at the University of Milano-Bicocca (Department of Earth and Environmental Sciences) and the second one for the three non-enzymatic biomarkers measured at the University of Milan (Department of Environmental Science and Policy). The two Universities received four samples for each colony, stage, treatment and date (864 samples in total) which were analyzed singularly. For each worker bee sample (single specimen), AChE activity was analyzed in the head portion of the body after dissection, and CAT, GST and ALP activities in the thorax without wings and in the abdomen (joined together); these three activities were analyzed starting from the same extract. AChE activity was analyzed in the head because of the highest content of nervous tissue, while CAT, GST and ALP were analyzed in the abdomen because of the presence of most of the digestive tract, which is primary involved in contaminant effects coming from the diet. The thorax without wings was added to have high amount of extract to perform on the same specimen the three enzymatic assays, according to previous analyses [11]; nevertheless, thoraxes have high chitin content. For this reason, on the second set of samples for the analysis of ROS, LPO and DNAFRAGM biomarkers, worker bee sample (single specimen) were analyzed in the head and abdomen (joined together), excluding thorax. In this case it was not necessary to add thorax to the extracted sample, because the head could have been joined to the abdomen portion. All the enzymatic determinations were done at least in duplicate, and the mean was considered as final data. As laboratory practice, intra-day and inter-day variability was assessed repeating, on the same day and in different days during the analysis of the whole data set, the same determination on random samples. Intra-day and inter-day mean variability (variation coefficient of 8.1% and 10.8%, respectively) were used to define overall precision.

Full methodology is available in Supplementary Materials of the online version of this article. Enzymatic biomarkers (AChE, CAT, GST and ALP) were expressed as international units (IU) in μmol min^−1^ mL^−1^ and referred to protein concentration (mg mL^−1^), resulting in the unit of μmol min^−1^ mg^−1^ of protein. ROS content was expressed as arbitrary units of fluorescence (AU) normalized to extracted fresh weight (AU g^−1^ f.w.). LPO was expressed as nmol TBARS formed per g of extracted fresh weight (nMol g^−1^ f.w.). DNA_FRAGM_ was expressed in μg of fragmented DNA per g of extracted fresh weight (μg DNA_FRAGM_ g^−1^ f.w.). Full methodologies are available in Supplementary Materials of the online version of this article.

### 2.3. Electric and Magnetic Field Measurements 

The presence of high-frequency electromagnetic field (HF-EMF) sources, in the frequency range of 100 kHz–2.5 GHz, was monitored by a Chauvin Arnoux C.A. 43 field meter. Low frequency electromagnetic fields (ELF-EMF) of 5Hz–100 kHz were measured with a tri-axial field analyzer PMM 8053 coupled with a PMM EHP 50A probe. On every sampling date of the biomarker monitoring in 2017, in the multi-stress site where a high-voltage transmission line was present (220 kV and frequency of 50 Hz, called Edis Tavazzano-Colà Me n° 220), 24 h measurements of the electric and magnetic fields were performed at 1.5 m above ground level where the hives were settled, with a time-resolution of 5 min. In addition, to characterize the decreasing gradient as a function of the distance, measurements of the magnetic and electric fields were performed at the same height at various distances from the transmission line. 

### 2.4. Exposure to Pesticides

Pesticide- and multi-stress sites were located inside an experimental orchard farm intensively cultivated by the following crops: apple (1.5 ha), pear (1.0 ha), peach (5.0 ha), apricot (0.2 ha), plum (1.5 ha), cherry (0.15 ha) and small fruits (0.15 ha). The complete schedule of the pesticide treatments in 2017 was obtained by the farm director and included dates and amounts of commercial products used on each crop. From the active ingredient content of each commercial product, the amount of active ingredients used in the farm was derived. Additional exposure to pesticides from the surrounding agricultural area, where mainly arable crops (maize and soybean) are present, was deduced by information obtained by the local agricultural consortium (https://terrepadane.it/, accessed on 28 January 2021) which is the main supplier of seeds, fertilizers and pesticides to local farmers.

### 2.5. Meteorological Data 

Daily mean temperatures with minimum and maximum hourly mean values and cumulated daily precipitations were obtained by the meteorological network of the Regional Environmental Protection Agency (https://www.arpalombardia.it/Pages/Meteorologia/Richiesta-dati-misurati.aspx, accessed on 20 January 2021). Two meteorological stations were selected in order to compare the two experimental locations (control and exposure sites): “Rivolta d’Adda” and “Cavenago d’Adda”, 3 km East from the control site and 14 km South-East from the exposure sites, respectively. Data were taken from 1 January 2017 until 30 June 2018.

### 2.6. Statistical Analyses

Biomarker activities were analyzed after decimal Log transformation, because of the significant shift from normal distribution, considering all data (n = 864 as maximum) and each developmental stage separately (n = 432 as maximum) (Kolmogorov-Smirnov test, *p* < 0.002). On the contrary, Log-transformed data, especially within each site (n = 144 as maximum), approached normal distribution (*p* > 0.05). Outliers, identified by box-plot analysis, were reduced to few cases, not excluded by graphical and statistical analyses because they were near the edges of the distribution boxes.

Generalized Linear Models (GLM) of Log-transformed data were performed, using enzymatic activities as dependent variables, and “hive”, ‘date’, ‘developmental stage’ and ‘treatment’ as factors. Tukey’s post-hoc test was used to establish significant differences between groups. Correlation analyses were performed using Pearson’s coefficient. Box plot and statistical analyses were performed using the program SPSS v. 15.0 (IBM SPSS Statistics, Armonk, NY, USA).

## 3. Results

### 3.1. Exposure to Pesticides 

Chemical treatments in the orchard farm potentially impacted experimental hives by residues in the air, vegetation and surface-water compartments. Their role in exposure to pesticides was of primary importance. Pesticide list and treatment schedule are reported in Table 2 and Figure S5, respectively. The number and amount of treatments was quite impressive: 33 different active ingredients (15 and 18 fungicides and insecticides, respectively) and 111 treatments (67 and 44 with fungicides and insecticides, respectively) with a total amount of active ingredients of 268 kg (150 and 118 with fungicides and insecticides, respectively) on 9.35 ha of cultivated area (28.6 kg/ha). Three quarters of them were mineral oil (111 kg), sulphur (50 kg) and copper hydrochloride (45 kg). Many insecticides were used and they included insecticides characterized by different modes of action: the neonicotinoids imidacloprid and thiametoxan, the organophosphates chlorpyrifos-methyl and phosmet, the pyrethroids deltamethrin, etofenprox and tau-fluvalinate, the ossadiazine methoxyfenozide, the ryanoids chlorantraniliprole, the spynosin spinosad, the avermectin abamectin, and the chitin synthesis inhibitor, triflumuron. 

On maize, cultivated in the surrounding, insecticides can be used during sowing as seed tanning between March and April. Seeds treated with neonicotinoids were forbidden since 2008, instead of them mainly pyrethroids, such as tefluthrin, are currently used (e.g., 5 g of tefluthrin/ha). Furthermore, during the growing season, insecticides can be used against the Lepidopteran *Ostrinia nubilalis* and the Coleopteran *Diabrotica virgifera* but their occurrence is limited. On soybean, mainly herbicides are used both in the pre- and post-emerging phase, while miticides against the mite *Tetranychus urticae* are occasionally used. However, we cannot totally exclude these possible sources of pesticide, as orchard crops with their blossoming period and the proximity to the colonies were considered to provide the main source of forage in the area; therefore, they were also considered to be the main source of exposure to pesticides in the pesticide and multi-stress sites.

### 3.2. Magnetic and Electric Field Measurements

**Figure 2:**
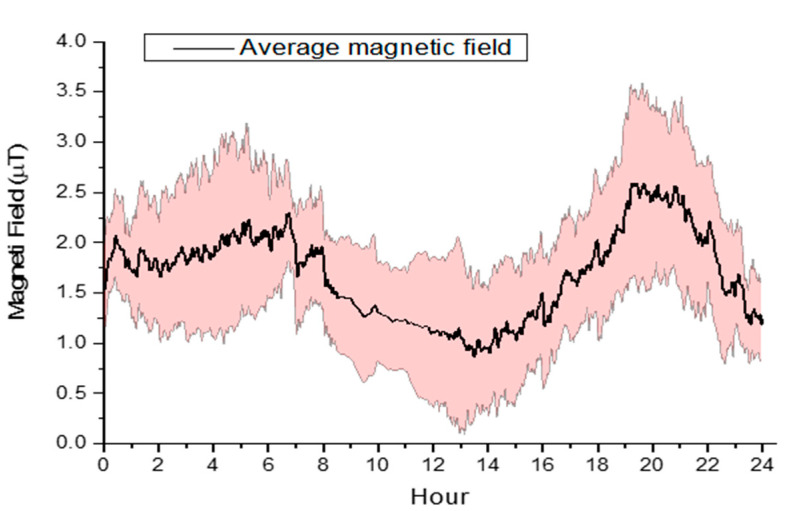

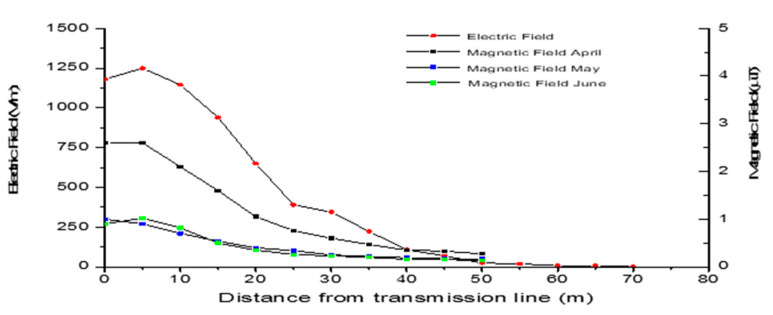
Mean daily cycle of the magnetic field below the transmission line (**above**) and the decreasing gradient of the magnetic and electric fields as a function of the distance from the transmission line (**below**).

Measurements of high-frequency electromagnetic field (HF-EMF) revealed no significant differences in all sampling sites. In control and pesticide-stress sites, measurements of extremely-low-frequency electromagnetic field (ELF-EMF) revealed background levels, while in the multi-stress site, at the hive level (below the high-voltage transmission line), the electric field was almost constant with an intensity of 1250 V/m (Figure 2). The electric field is mainly determined by the distance from the transmission line and the characteristics of the transmission line itself (voltage) and therefore it is almost constant, while the magnetic field depends on the line load (electricity demand associated to the request of human activities), and therefore it is subject to variable daily cyclic variations (Figure 2 above). During the field trial, the magnetic field was 1.49 ± 0.65 μT (mean ± standard deviation) with a mean daily peak intensity of 2.43 ± 0.97 μT (mean ± standard deviation). The decreasing gradient of the magnetic and electric fields as a function of the distance from the transmission line is shown in Figure 2 (below).

### 3.3. Meteorological Conditions in the Experimental Sites

Meteorological data are reported in Figure S7. The two meteorological stations (“Rivolta d’Adda” and “Cavenago d’Adda”), selected for the experimental sites, are located 3 km East from the control site and 14 km South-East from the exposure site, respectively. Due to the proximity of the two meteorological stations (22 km), mean temperature and cumulative precipitation daily data were very similar and highly correlated (r = 0.998, n = 538, *p* < 0.001 *** and r = 0.874, n = 546, *p* < 0.001 ***, respectively). Considering that the two experimental sites were even nearer (15 km) than the two meteorological stations (22 km), their meteorological conditions could also have been more similar.

### 3.4. Health Status of the Colonies

During the trial, colony survival, queen activity, storage and brood amount and presence of parasites and pathogens were checked regularly on each hive. Family survival greatly varied depending on sites: on 15 June 2017, in the multi-stress site a colony (MU-2 hive) was destroyed following the discovering of American foulbrood (*Paenibacillus larvae*). On 29 June 2017, two other families (CH-1 and CH-3 hives) in the chemical-stress site were affected by American foulbrood but, instead of destroying them, an antibiotic treatment with Oxytetracycline Hydrochloride (≥98%, Sigma Aldrich, Darmstadt, Germany) was planned on all the experimental hives in order to stop the disease, save the trial and maintain the comparability among sites. On 3 July, antibiotic treatment in sugar solution (1 mg/mL) was applied on the top of honeycomb of each hive (5 mL per comb, 0.05 g of antibiotic per hive) then a second and a third antibiotic cycle was repeated at a one week interval (10 and 17 July). During the third antibiotic cycle a colony in the multi-stress site (MU-4 hive) was almost lost and severely infested by the larvae of the lepidoptera *Galleria mellonella*. For this reason, it was destroyed. On the contrary, in the chemical stress-site the two hives affected by American foulbrood 20 days before were in a good health state, as were those in the control site, where no American foulbrood was detected. On 14 September in the control site a colony (CTL-2 hive) was lost by depopulation: only the queen and few workers were present concomitantly with a severe infestation by *Galleria mellonella* larvae. This depopulation event was related to deposition of only drone brood by a new queen purchased in the market and introduced in the hive on 21 July after the detection of queen absence (due to the death probably during the mating flight of the newly emerged queen observed on the honeycombs 14 days before) and as eggs and few three-day-larvae were not present to allow natural replacement. The depopulation probably started in August, when the eldest workers began to die and were not replaced by the new ones, due to male brood deposition by the new queen, but this was not immediately evident until the end of August. Male brood deposition was soon observed, but the oviposition of sterile eggs also in the worker cells was initially misunderstood. Only later on was the male deposition evident concomitantly with the low number of workers, giving in September a condition of not recoverable. In all other hives, after the brood interruption, the deposition carried on almost regularly also in exposed sites. On 15 November 2017, in multi-stress site, another colony (MU-1) was lost by depopulation; in this hive 11 October 2017 few brood and two queens were present and on 31 October 2017 no brood at all was detected. At the end of the trial (17 April 2018), out of the four families present in each experimental site, three survived in the control one, four in the chemical-stress one and only one in the multi-stress site.

After the diagnosis, in mid-June, one case of American foulbrood samples of powdered sugar and debris previously collected and stored for *Varroa* monitoring were examined for *P. larvae* detection. *P. larvae* genotype ERIC II was found in the hive debris collected under the hives. At the end of April, colonies that later became ill showed already a high number of *P. larvae* spores. The powdered sugar samples collected at the end of May in the two stress sites revealed that *P. larvae* ranged from 174,000 to 5,000,000 CFU/g in diseased colonies and from 60 to 17,000 CFU/g in the asymptomatic ones, whereas in the control site, *P. larvae* was detected only in one sample (detection limit: 20 CFU/g). 

Virus infections were analysed in dead bees collected from the underbaskets. Acute bee paralysis virus (ABPV) was found in almost all samples, mainly at low levels of infection, as likely expression of latency, and just in two cases with values up to 9.9 × 10^8^ in two colonies in the multi-stress site, respectively, in September and October 2017. Deformed wing virus (DWV) was found in fewer samples, again mostly at low level of infection, and just in three cases at relevant levels of infection. In the multi-stress site, it was detected in the same two colonies that resulted positive for ABPV at 1.4 and 5.6 × 10^5^ viral genome copies per bee, respectively. However, DWV was also related to *V. destructor* infestation since it was detected at 1.1 × 10^5^ viral genome copies per bee also in a control hive where a high level of *V. destructor* was present (CTL-2 hive), the one which was then lost by depopulation on the 14th of September.

**Figure 3:**
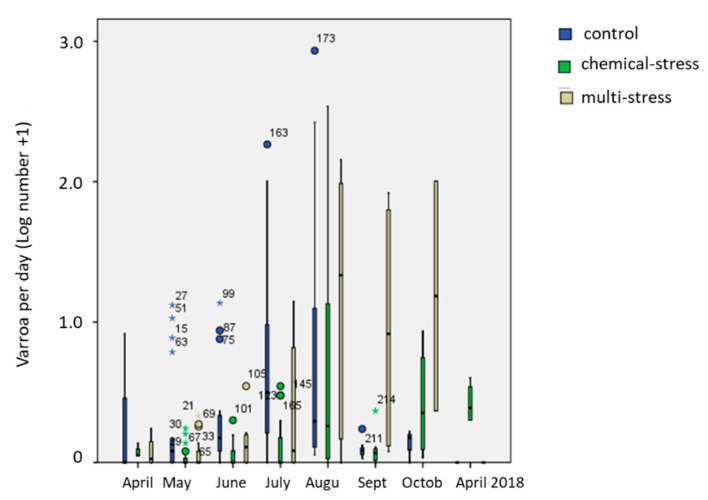
Box plot of fallen varroa mites per day (decimal logarithm of the mite number +1) in the experimental sites from April 2017 to April 2018. Circles indicate data faraway less than 3 times the interquartile distance; stars data faraway more than 3 times the interquartile distance.

*Varroa destructor* monitoring was performed, counting naturally fallen mites on an adhesive sticky board changed at each visit. Plotted data in Figure 3 were Log-transformed because of the high variability between sites, dates and hives. Maximum number of fallen mites per day was 857, 342 and 143 in the control, chemical- and multi-stress sites, respectively, while the median values were very low for all (0.40, 0.08 and 0.20, respectively). GLM on Log-transformed data showed that week of sampling was highly significant (F_21;209_ = 6.0; *p* < 0.001 ***) as well as the intra-treatment variability (F_3;209_ = 4.5; *p* = 0.004 **), confirming that varroa mite infestation was irregularly but equally distributed among exposure and control sites, depending on the season (“date” factor) and on the single-hive history (“hive” factor). Treatment factor was significant too (F_2;209_ = 6.7; *p* = 0.002 **), but differences among sites were highly dependent on “date” and “hive”. *Varroa* was already abundant, in one of the control hives (CTL-3 hive) at the beginning of the trial (uncontrolled variability). During the trial the maximum number of fallen mites per day was reached in August after the anti-varroa treatment (2 August), consequently, in September, *V. destructor* presence was very low in all hives with the exception of one hive in the multi-stress site (MU-1 hive) which persisted to have a high varroa mite infestation also in October. The same hive was lost the 15th of November by depopulation. 

Varroa mite monitoring by powdered sugar application was additionally performed on four dates in all experimental hives. Fallen mites recorded after 1 h from the application were highly correlated to the data of naturally fallen varroa mites (Pearson’s correlation coefficient, r = 0.99; n = 40; *p* < 0.001 ***).

Anomalies in queen activity were detected, especially in the multi-stress site, where in 2017 a total of six queen changes were recorded (three in MU-1 and three in MU-4 hives); in the other sites (control and chemical-stress), three queen changes each were recorded (one and two queen changes in CTL-1 and CTL-2 hives and two and one in CH-2 and CH-4 hives, respectively). Most of the queen changes happened between 24 May and to 27 July 2017, and they were linked to new queen rearing and, as cited above, a case with two queens contemporarily present was observed (MU-1 hive). 

**Figure 4:**
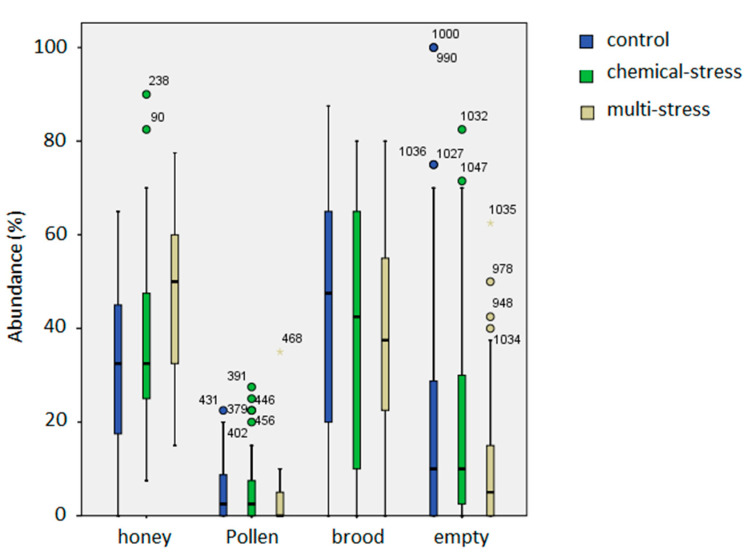
Box-plot chart of the honeycomb occupancy by honey, pollen, brood and empty space in the three experimental sites.

Honeycomb occupancy was constantly evaluated from April to November 2017 and again in April 2018, to assess the amount of storages (honey and pollen), brood presence (new and operculated) and unused space (empty honeycombs). Drone brood was recorded but it was not considered in the analyses because it was present irregularly and in low amount, except in a few cases, as mentioned above (CTL-2 hive in August 2017). Honeycomb occupancy in each hive is shown in Figure 4 by the box-plot analysis of the mean percentage of honey, pollen, brood and empty space occupancy in every hive during the whole trial (12 hives in the three experimental sites × 25 sampling date = 300 data as maximum). 

Honey, pollen, brood and empty space, as percentage of occupancy, were tested by GLM analyses in relation to “treatment”, “date” and “hive” as factors (Table 3). The “date” factor accounts for the seasonal variability in the honeycomb occupancy and it was significant for all the considered variables (honey, pollen, brood and empty space). The “hive” factor accounts for the intra-treatment variability, and it significantly affected honey and empty space, while the “treatment” factor was significant for honey, pollen and brood amount (*p* < 0.001 ***, *p* = 0.022 *, *p* = 0.022 *, respectively). 

The multi-stress site showed a higher accumulation of honey in respect to the other sites and a lower amount of stored pollen and empty space, as confirmed by post hoc analysis (Tukey, *p* < 0.001 **). The lower amount of brood in chemical and multi-stress sites (Figure 4) was not significant by the Tukey test (*p* > 0.05), even if GLM analysis was significant for “treatment” factor (GLM, *p* < 0.05). Honey and pollen in the chemical site were statistically different from that in the multi-stress site but not from that in the control one (Tukey, *p* < 0.05).

**Figure 5:**
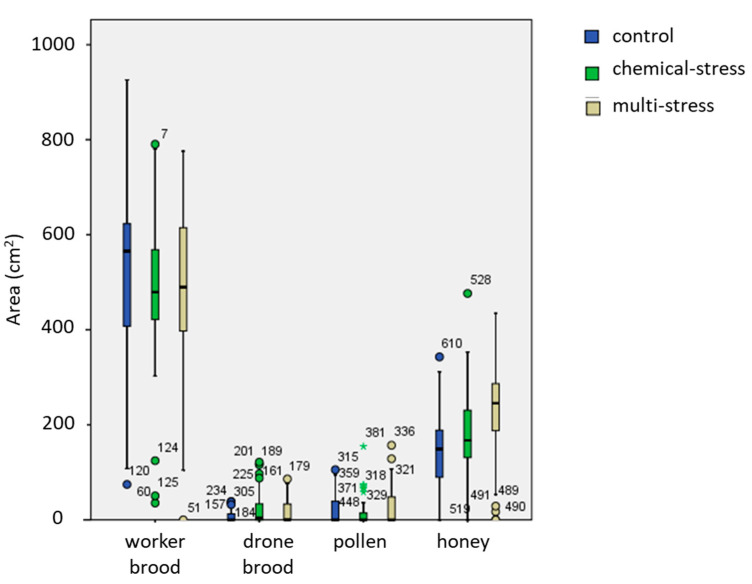
Box-plot chart of the area occupied by worker-brood, drone-brood, pollen and honey in the central honeycomb in the three experimental sites.

These results were confirmed by the central-comb analysis of each hive (Figure 5). Higher accumulation of honey in the multi-stress site and a lower amount of worker-brood was evident, as well as the intermediate condition of the chemical-stress site. Statistical analyses by GLM confirmed significant effect of the “treatment” factor for honey and drone-brood (GLM: F_2;118_ = 10.2; *p* < 0.001 ***; F_2;118_ = 2.7; *p* = 0.002 **, respectively). The multi-stress site showed a higher accumulation of honey (Tukey test, *p* = 0.0185), while the honey amount in the chemical site was statistically different from that in the multi-stress site but not from that in the control (Tukey test, *p* > 0.05). Marginal mean value of honey area was 230 cm^2^ in multi-stress site and 171 cm^2^ and 142 cm^2^ in the chemical and control sites, respectively. Drone-brood area was significantly higher in the chemical and multi-stress sites (Tukey test, *p* = 0.001 **, marginal mean values of 25 cm^2^ and 6.9 cm^2^, respectively). 

Health status of the colonies was monitored also by collecting dead specimens in front of each hive (mortality in the underbaskets, Table 4). Mean number of dead worker bees were higher in treated sites in comparison to those in the control. Deformed worker bees, counted separately, were higher in the control site, due to the detection of a high level of infection with DWV in one hive of the control site. Drones and pupae were not different among sites, while the number of dead queens was higher in the multi-stress site. Queen and pupae (drones and worker bees) were detected rarely, and deformed drones were more frequent than deformed worker bees in relation to normal ones. 

**Figure 6:**
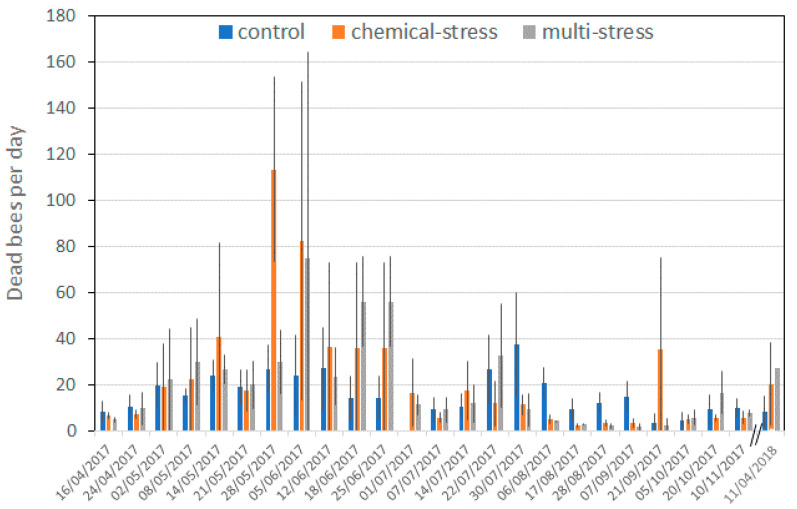
Mean number of dead worker bees per day in the control, chemical- and multi-stress sites as a function to the date of sampling.

The mean number of dead worker bees per day in the three experimental sites is reported in Figure 6. 

Statistical analyses were performed only on normal worker bees, because of their higher number in the underbaskets. GLM was applied after transforming the “dead worker bees” variable by logarithm, because of the shift from normal distribution of the non-transformed data (z = 3.6; n = 266; *p* < 0.001 and z = 0.71; n = 266; *p* = 0.69 for non-transformed and transformed data, respectively). GLM considering “dead worker bees” as dependent variable and “treatment”, “period” and “hive” as factors, revealed that “hive” and “period” significantly affected mortality in the underbaskets (F_3;236_ = 5.96, *p* = 0.001; F_24;236_ = 5.33, *p* < 0.001, respectively), the “treatment” factor was just above the significant threshold (F_2;236_ = 2.59; *p* = 0.077), while the interaction “treatment” × “period” was highly significant (F_47;192_ = 2.5; *p* < 0.001), evidencing that in chemical stress site especially and in multi-stress sites, several peaks of mortality occurred in particular at the beginning of June (62, 42 and 20 dead worker bees per day—geometric marginal means—in the chemical, multi-stress and control sites, respectively).

### 3.5. Biomarker Analysis

#### 3.5.1. Biomarkers in the Control Site

**Figure 7:**
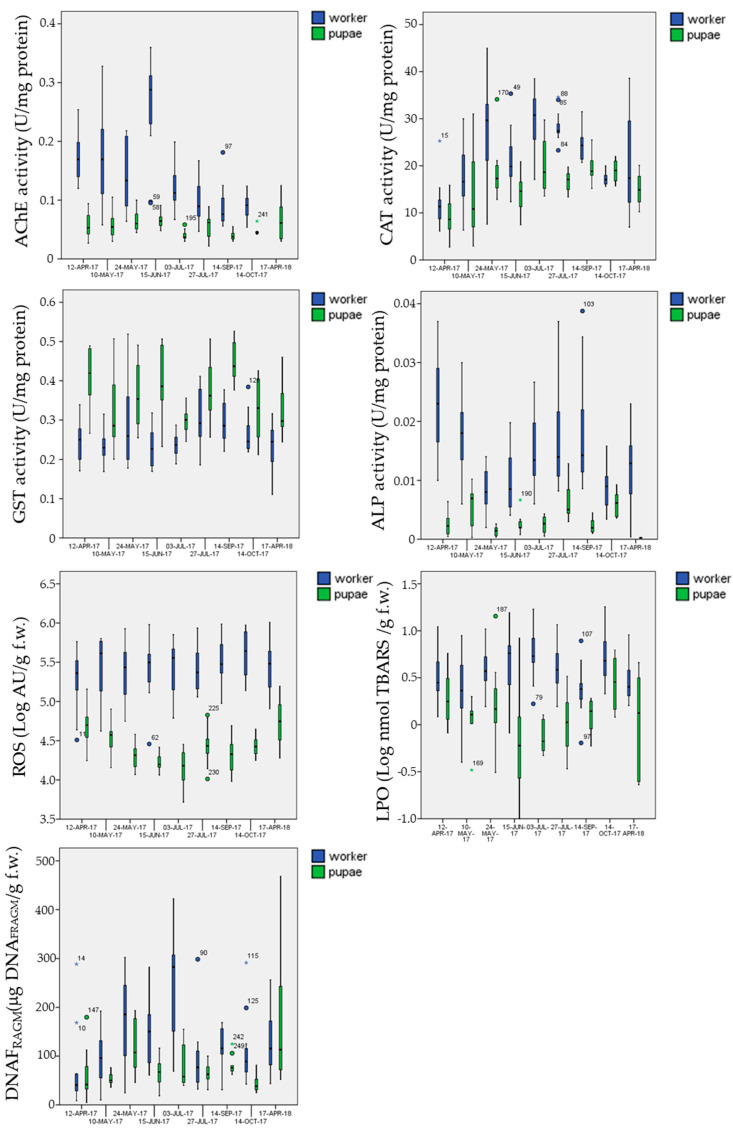
Box-plots of the analyzed biomarkers in the control site as a function to the sampling date.

Biomarker results in the control sites can be considered representative of the physiological conditions. Considering that an entire year cycle (from April to April) was analyzed, they can be considered of high interest from a methodological point of view. For all biomarkers, except GST, mean and percentiles values were higher in worker bees with respect to the pupal stage (Table 5). The box plot analysis in Figure 7 also highlights a clear seasonal trend, such as for CAT with lower value in the spring and autumn and higher ones in summer. Activation of CAT in summer seems to offset the ROS levels, which were quite constant in worker bees and in larvae during the year. 

Statistical analyses were performed on Log-transformed data, because of the shift from the normal distribution, and evidenced that the “stage” factor was highly significant for all biomarkers (F_1;195__–233_ > 29; *p* < 0.001 ***), the “date” factor, even if less evident, was significant for all biomarkers (F_8;221__–233_ > 2.5; *p* < 0.012), except LPO (F_8;195_ > 1.6; *p* ≤ 0.14), and the “hive” factor, which accounts for intra-control replicates, never had a significant effect (F_3;195__–233_ < 1.6; *p* > 0.14) or was near the significative level (F_3;221__–233_ < 2.6; *p* > 0.052).

#### 3.5.2. Biomarkers in the Exposure Sites

**Figure 8:**
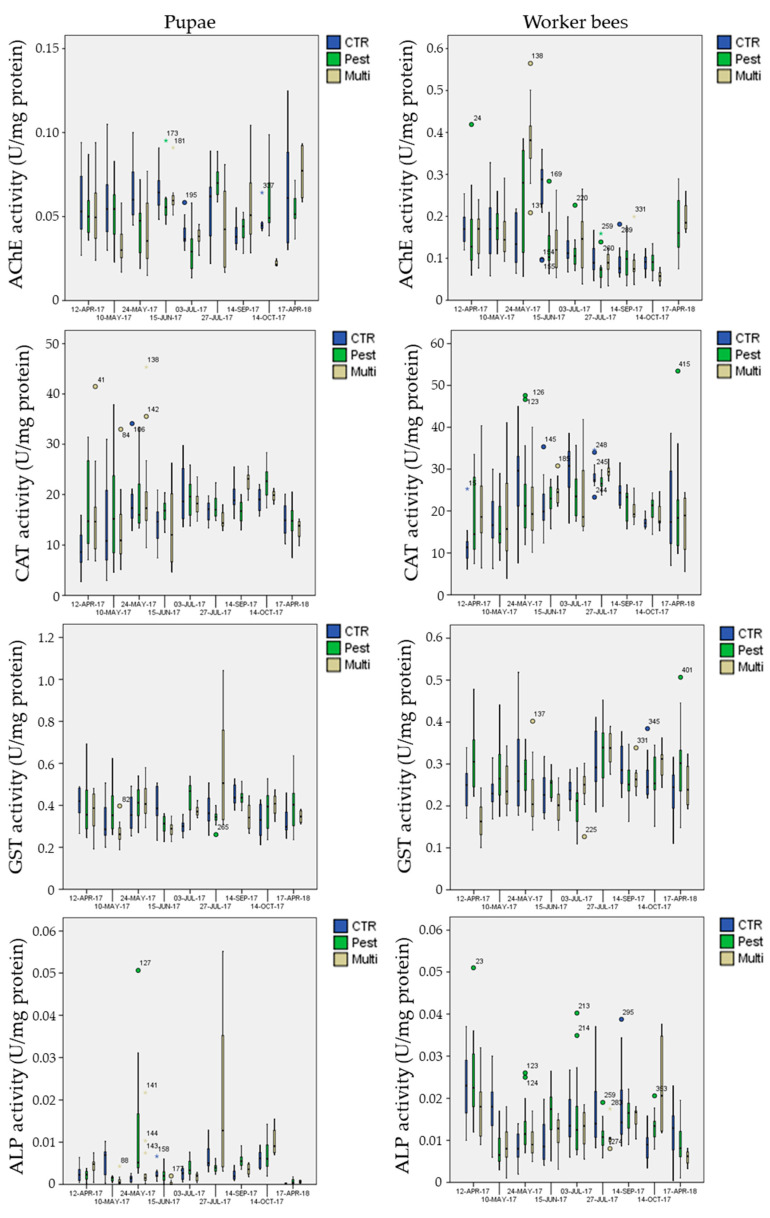
Box-plot of AChe, CAT, GST and ALP activity in the three experimental sites for pupae and worker bees as a function of the sampling date.

**Figure 9:**
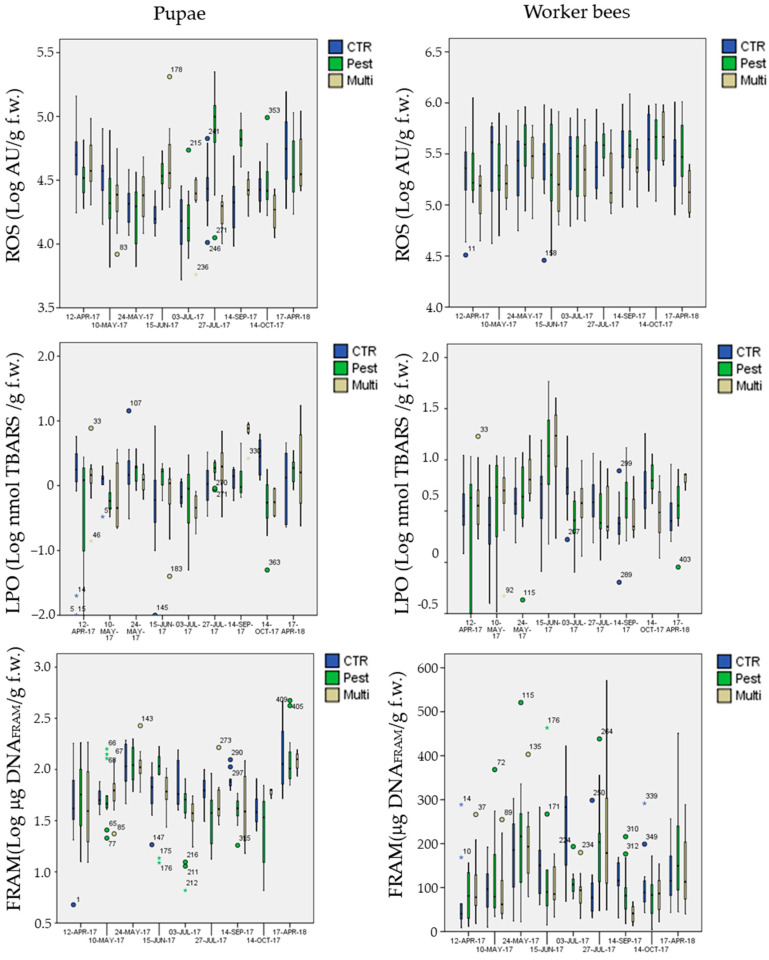
Box-plots of ROS, LPO, DNA_FRAGM_ in the three experimental sites for pupae and worker bees as a function to the sampling date.

Considering the different behavior between the two stages, biomarker levels in exposure sites were analyzed separately for pupae and worker bees (Figure 8 and Figure 9). Results of the GLM, performed on Log-transformed data, are reported in Table 6.

Few biomarkers presented significant mean differences among the treatments, independently of the date of sampling (“treatment” factor in Table 6):

- mean AChE activity in pupae was significantly inhibited (Post-hoc Tukey test *p* = 0.002 **) in the multi-stress site (marginal mean of 0.041 U mg^−1^ protein with 95% confidence Interval of 0.037–0.045) in comparison to the chemical-stress (marginal mean of 0.050 U mg^−1^ protein, CI = 0.046–0.053) and control sites (marginal mean of 0.051 U mg^−1^ protein CI = 0.048–0.055);
- mean CAT activity in pupae was significantly activated (Post-hoc Tukey test *p* < 0.023 *) in the chemical-stress site (marginal mean of 17.0 U mg^−1^ protein, CI = 16.0–18.0) in comparison to the control (marginal mean of 15.2 U mg^−1^ protein, CI = 14.2–16.3) and multi-stress sites (marginal mean of 15.6 U mg^−1^ protein CI = 14.3–17.0);
- mean GST activity in pupae was significantly activated (Post-hoc Tukey test *p* < 0.030 *) in the chemical-stress site (marginal mean of 0.38 U mg^−1^ protein, CI = 0.36–0.39) in comparison to the control (marginal mean of 0.36 U mg^−1^ protein, CI = 0.34–0.37) and multi-stress sites (marginal mean of 0.36 U mg^−1^ protein CI = 0.34–0.38), while in worker bees mean GST activity was higher in the chemical-stress site (marginal mean of 0.27 U mg^−1^ protein, CI = 0.26–0.28) in comparison to the control (marginal mean of 0.25 U mg^−1^ protein, CI = 0.24–0.26) and multi-stress sites (marginal mean of 0.24 U mg^−1^ protein CI = 0.23–0.25), but differences were only significant with the multi-stress site (Post-hoc Tukey test *p* = 0.003 **);
- mean ROS levels in pupae were significantly higher (Post-hoc Tukey test *p* < 0.003 **) in the chemical-stress site (marginal mean of 3.3 × 10^4^ AU g^−1^ f.w., CI = 3.0 × 10^4^−3.5 × 10^4^) in comparison to the control (marginal mean of 2.6 × 10^4^ AU g^−1^ f.w., CI = 2.4 × 10^4^−2.9 × 10^4^) and multi-stress sites (marginal mean of 2.6 × 10^4^ AU g^−1^ f.w., CI = 2.3 × 10^4^−3.0 × 10^4^), in worker bees ROS levels were higher in the chemical-stress site (marginal mean of 3.0 × 10^5^ AU g^−1^ f.w., CI = 2.6 × 10^5^−3.4 × 10^5^) in comparison to the control (marginal mean of 2.7 × 10^5^ AU g^−1^ f.w., CI = 2.4 × 10^5^−3.1×10^5^), but differences were not significant (Post-hoc Tukey test *p* = 0.39); the multi-stress site showed a mean lower level of ROS (marginal mean of 2.0×10^5^ AU g^−1^ f.w., CI = 1.7 × 10^5^−2.4 × 10^5^) in comparison to the control and chemical stress sites (Post-hoc Tukey test *p* < 0.021 *);
- mean DNA_FRAGM_ levels in pupae were significantly higher (Post-hoc Tukey test *p* < 0.006 **) in the control site (marginal mean of 66 μg DNA_FRAM_ g^−1^ f.w., CI = 59−74) in comparison to the chemical-stress site (marginal mean of 53 μg DNA_FRAM_ g^−1^ f.w., CI = 48–59); the multi-stress site was inbetween (marginal mean of 59 μg DNA_FRAM_ g^−1^ f.w., CI = 50−69).

Data*treatment interaction was significant for all biomarkers and life stages (Table 6, GLM; *p* < 0.015 *), except for ROS levels in worker bees (GLM; *p* = 0.71); post-hoc Tukey test (*p* < 0.05 *) for all sampling dates, biomarkers and life stages revealed that 61 exposure vs. control comparisons of 126 (48%) were significant (51% and 46% in pupae and worker bees, respectively). 

Generally, most of the significant differences were observed in summer and autumn sampling, while in April 2017 and April 2018 most of the biomarkers in exposure sites were not different from those in the control. In many cases, the chemical- and multi-stress sites showed a similar effect in respect to the control, such as on 24 May when an evident AChE inhibition was observed in pupae or on 15 June in worker bees (Figure 8), while in other cases a significant effect was observed only in the multi-stress site (AChE inhibition 10 May in pupae) or in the chemical-stress site (AChE inhibition 27 July in worker bees). 

## 4. Discussion

The fundamental role of pollinators for agriculture and biodiversity requires the evaluation of stress sources for honeybees, especially in field conditions. A recent review [58] highlighted that most of the studies on sub-lethal effects on pollinators were performed in Europe and North America, and most of the studies were addressed to insecticides and they were conducted in laboratory under controlled conditions. The importance of testing real field conditions is well recognized for evaluating the complexity of environmental factors and possible stress sources. Although highly recommended for ecological realism, this approach presents several problems in the definition of the reference conditions (control), in the interpretation of the results (direct cause-effect identification) and in the possibility of uncontrolled events. The loss of one hive in the control site resulted in a quite high mortality because of the low number of replicates (1 of 4, 25%), but we evaluate that this collapse was not due to the site characteristics (control) but to an external event due to a new queen (declared mated but unfortunately virgin) coming from market following the loss of the pre-existing queen during the mating flight. The previous queen, possibly changed for an effect of the site, was therefore considered when queen changes among treatments were evaluated. As a general observation, the low number of hives in each experimental site (n = 4) is an objective limitation, especially from a statistical point of view, but the complexity of the study and the high number of parameters analyzed on each hive imposed this restrictive choice. However, the information coming from the present research is complex and despite the low number of colonies it can give a picture of possible responses of colonies subjected to the effects of different stresses in field. 

In the control site, in one colony high levels of DWV were detected concomitantly with a high level of *Varroa* infestation. The association between varroa numbers and virus transmission is documented in literature (e.g., [65]). This happened only in one hive, while the others were in good health during the whole trial: low *Varroa* infestation, low viruses detection, low mortality in the underbaskets, low queen changes and anomalies, equilibrated storage accumulation and brood deposition. In this site, differently from the exposure ones (chemical- and multi-stress), no American foulbrood disease was detected, even in the hive severely affected by varroa mites and DWV (CTl-2). On the contrary, in June 2017 one case of American foulbrood was detected in the muli-stress site and later other two cases in the chemical-stress one. The analysis of *P. larvae* spores in hive debris collected from the beginning of the trial revealed that *P. larvae* spores grew up in the hives where the disease occurred, but they were also present at low levels in other exposure hives and even in one hive of the control site (CTL-1). The origin of the hives was homogeneous (same producer) and thus it was not surprising that the pathogen spores could have been present in all hives independently from the treatment. Therefore, we hypothesize that low contamination was present at the beginning of the trial in all the colonies, and that the presence of stressors in exposure sites probably favored the development of the infection, increasing the number of spores and giving rise to the disease. This hypothesis is supported by the results obtained by [66] in a laboratory test. In this work the combined effect of pesticides and *P. larvae* infection on larval mortality was studied and a synergistic interaction between a *P. larva* genotype ERIC II and different classes of pesticide in co-exposed larvae was demonstrated. Either the organophosphate dimethoate or the neonicotinoid clothianidin fed in sublethal doses to larvae previously infected with AFB significantly elevated larval mortality. It is interesting that in the control site, even if presumably present, *P. larvae* spores did not grow up and the symptoms did not occur. On the contrary, the disease happened in both exposure sites (chemical- and multi-stress), concomitantly with the detection of a high number of spores in the hive debris. In literature, the detrimental effect of pesticides on the immune system of bees is well known [41]. Beside American foulbrood occurrence in the pesticide- and multi-stress sites, all the other health status parameters indicated that exposure sites were subject to significant stress sources: mortality in the underbasket, especially in May and June, showed higher levels of worker bee and queen mortality; a higher frequency of queen changes was detected in the multi-stress site together with a higher amount of honey storage and a lower amount of brood deposition. Fallen varroa mites were high in one hive of the control site (CTL-2), as mentioned above, but they were also particularly high in autumn in the multi-stress site. The final survival of just one colony in the multi-stress site was the striking fact of the anomalies and the problems that occurred in the bee colonies at this site.

The use of a battery of biomarkers on two life stages (pupae and worker bees) allowed the study of these parameters in “stress conditions” (exposure sites) but also in “physiological conditions” (control site). In the literature, biomarkers were already used to test different stresses (urban pollution and pesticides) on *Apis mellifera* both in the field [67,68,69,70,71,72] and in laboratory [73,74,75]. In the field, a reference station is always chosen away from anthropogenic sources but close enough to the experimental sites to have a direct comparison [68,69,70,74]. All the literature accords to the need of taking into account both the seasonal [11,68,76,77,78] and the physiological variability of biomarkers in relation to the life stage, age and sex [79,80,81,82]. For example, enzymes involved in the metabolism of many xenobiotics, such as the cytochrome P450 complex in the pupae, are not yet as developed or efficient as in the adult bee [83]; for this reason, [84] underlines the need to study the toxicity of pesticides (acute and chronic) in the preimaginal stages instead of the adult one, as actually required by the European legislation [85].

In this work, in the control site biomarker results, pupae and adult bees were significantly affected by the life stage and the date of sampling. Biomarker values obtained in the pupae were always lower than those measured in worker bees; only the enzymatic activity of GST was higher in the pupal stage than in the adult bees, according to what is observed in other species [86]. A greater expression of some classes of genes that encode for isoforms of GST enzyme in the larvae and pupal stage of insects and arthropods was reported in literature [11,87,88]. Lupi et al. [11] discussed the relationship between temperature (seasonality) and enzymatic activity of in-hive worker and forager bees, concluding that biomarker levels are probably more related to the physiological cycle of the hive activities (high in spring and summer and low in autumn) than directly to the annual temperature cycle. In spring and summer, the hive activity of both food recruitment and brood rearing is particularly high, on the contrary, in autumn bee colonies slow down their activities because they prepare themselves for overwintering. The present results seem to accord to Lupi et al.’s hypothesis, at least for some biomarkers. For example, AChE activity showed a decreasing trend from April until November, with the maximum in June when the hive activities are at maximum. On the contrary, CAT activity seems to be more related to the annual cycle of temperatures than to the hive activity, both in pupae and in worker bees. In ectothermic animals, temperatures have a direct effect on metabolism and it is known that an increase in metabolism leads to an increase in ROS production [89] and, consequently, to the activation of antioxidant enzymes to compensate for the excess of ROS. When the antioxidant defenses are not sufficient, oxidative damages can occur, such as those to membrane lipids and proteins. In this work, LPO levels seem to be higher in summer than in spring and autumn, but this happened only in worker bees and not in pupae. Presumably, the activation of CAT activity in summer, determined by ROS overproduction in this period, was sufficient to neutralize oxidative stress in pupae but not in worker bees. It is evident that, during the seasonal cycle, the different stages (pupae and worker bees) respond differently to the different conditions; therefore, according to [73,77], biomarker activities appear to be related to a combination of climatic features, hive activities and physiological characteristics of the different life stages.

Exposure to pesticides, in Europe, is firstly evaluated by the hazard quotient (HQ) or Toxicity Exposure Ratio (TER) concept proposed by the EFSA Guidance Document on the risk assessment of plant protection products on bees (*Apis mellifera*, *Bombus* spp. and solitary bees) [90,91]. In analogy to the hazard quotient (HQ), which is the ratio between the quantity of active ingredient applied in kg a.i. hectare^−1^ and the toxicity on bees in μg bee^−1^ (contact or ingestion LC_50_ 48 h), pesticide stress was firstly evaluated by the Toxicity Ratio (TR) approach, which is the ratio between the applied amount of active ingredient in μg cm^−2^ and the toxicity on bees in μg bee^−1^ (contact or ingestion LC_50_ 48 h). The TR was preferred to the HQ because of the higher consistency of units (μg cm^−2^ and μg bee^−1^) and the more direct interpretation of the ratio. In fact, the final unit of bee cm^−1^ is roughly the inverse of the bee surface (1 cm^2^ bee^−1^) so that a TR higher than 1 means that the applied dose is able to produce a lethal effect on more than the 50% of the population, while a TR lower than 1 means that the applied dose is able to produce a lethal effect on less than the 50% of the population. The toxicity ratio (TR) was applied to the pesticides used in the area (Table S1 and Figure S6) and showed toxicity ratios ranging over more than 5 order of magnitude from 0.0019 for the plant regulator NAA (1-naphthylacetic acid) to 165 for the insecticide imidacloprid. By the TR approach (Figure S3) it is evident that from March until October, almost continuatively, very highly toxic insecticides (TR > 100) were applied in the orchard farm; they involve imidacloprid (TR = 165), thiametoxan (TR = 144), both neonicotinoid insecticides and the pyrethroid insecticide deltamethrin (TR = 133). In addition, several other toxic insecticides were applied with a TR between 10 and 100, such as chlorpyrifos-methyl (TR = 36) and emamectin benzoate (TR = 33) and with a TR between 1 and 10, such as spinosad (TR = 8.4) and etofenprox (TR = 9.2). Beside insecticides, fungicides were applied almost as regularly from March until October on the different crops. The number of fungicide treatments was even higher than that of insecticides (Table 2). Despite their low TR, their sub-lethal and chronic effects are extensively demonstrated by the literature (e.g., [92,93]). 

In the orchard farm, cultures flowered in March and April, directly attracting forager bees in that period, but, also later, bees can be attracted in the spraying area by the presence of natural vegetation among the trees (spontaneous herbaceous flowers) and around them (acacia and *Rubus* sp.). Forager bees are directly exposed to pesticides, passing outside the hive for up to 12 h a day; on the other hand, pupae and in-hive worker bees come into contact with pesticides mainly by residues transported and stored involuntarily inside the hive. In May and June, peaks of mortality were recorded in the underbaskets in the chemical- and in multi-stress sites (Figure 6) and the mean number of dead worker bees per day in the chemical- and in multi-stress sites (22.8 and 23.2 dead bees day^−1^, respectively) were higher than that in the control site (16.5 dead bees day^−1^). These events can be directly related to the exposure to pesticides, which was common to the two exposure sites. Moreover, mortality in the underbaskets accounted for only a part of the bee mortality and can be related more to pesticides residues transported into the hive. In the literature, exposure to pesticides is often evaluated through the analysis of pesticide residues in pollen and honey [94,95] or by more complex semi-quantitative indexes [96,97]. The authors of [22] reported a list of 34 pesticides found in wax, bees, honey and bee bread, coming from Italian colonies. Among those applied in the experimental area of the present research (Table 2), the insecticides imidacloprid, thiamethoxam and tau-fluvalinate were found by [22] in bee bread at the concentrations of 14−99 ng g^−1^, 14−1619 ng g^−1^ and 16−1537 ng g^−1^, respectively, and the fungicides pyrimethanil, cyprodinil, fludioxonil and tebuconazole at the concentrations of 18−584 ng g^−1^, 22−1560 ng g^−1^, 7−271 ng g^−1^ and 263 ng g^−1^, respectively, demonstrating that different active ingredients of both insecticides and fungicides may be found in the hive as residues in food storage. Moreover, pesticides are known to persist in honey, wax and pollen for a long time [98], extending the exposure period over that of the treatments. This makes impossible in the field the direct relationship between exposure (pesticide application) and toxicity, determining a shift in time and cumulative effects of the pesticide stress. 

It is difficult to explain the modulation of biomarker levels in terms of exposure to the stressors considered, as many and often unpredictable are the factors implied. Desmet et al. [99] evidenced discrepancies in immunosuppression and detoxification mechanisms between honeybees exposed to imidacloprid in laboratory cages and in-field honeybees. Field bees showed a more resilient response with an immune stimulation. For this reason, we can discuss the results only on a speculative way, comparing the results with other research conducted in laboratory or in field. We can consider the modulation obtained and the effects known in literature for bees and other organisms to justify our results and to compare the stressors. The inhibition of AChE activity in the highly stressed site is in line with the results of [71] where bees collected from agricultural areas shoved a reduction of AChe activity. However, it is strange that the same inhibition was not evidenced in the chemical-stress site. Furthermore, the major activation of mean CAT activity in pupae in the chemical-stress site in comparison to control is in line with the results in [100] where the activation of CAT was observed in the larvae of *Chironomus riparius* exposed to high concentrations of a pesticide. On the contrary, the lower activation of CAT in the multi-stress site compared to the chemical site could be due to the presence of electromagnetism, as evidenced in [101] where a reduction of CAT in mice exposed to radiation was evidenced. Moreover, as GSTs are a large family of multifunctional enzymes involved in the detoxification of a wide range of xenobiotics including insecticides, the significant activation of GST in both pupae and worker bees in the chemical-stress site compared to the control site is in line with what was expected. Different is the situation of the multi-stress site, where GST was not significantly different from the control site; however, this enzyme is extremely variable and different research has demonstrated its reduction in animals that were subjected to radiation [102,103]; as such, it could be possible that this result was a balance between the effect of pesticides and electromagnetism on bees. It is known from the literature that exposure to pesticides causes an increase in oxidative stress in numerous organisms [104,105,106], including bees [41,69,107,108], involving genetic damage too [105]. In the present study, ROS levels in pupae were found to be higher in the chemical-stress site than in the control, with the most evident ROS increase from June to September when most of the pesticides were applied. Surprisingly, ROS levels in the multi-stress site were often lower than in the pesticide-stress site and marginal mean levels in worker bees were even lower than those in the control site, showing a contrasting effect of the pesticide and electromagnetic exposure. Literature studies reporting an increase of oxidative stress caused by exposure to pesticides were carried out mainly with forager bees that have a more direct and acute exposure to pesticides. It is possible that the lower exposure of pupae and in-hive worker bees to pesticides could have reduced the evidence of such an effect in the present work. However, some sampling dates showed an evident increase of the lipid peroxidation in worker bees in the chemical- and multi-stress sites, such as the 15th of June and the 14th of September. DNA fragmentation presented contrasting results; in fact, besides sampling dates when it was lower in exposure sites than in the control (such as the 3rd of July for worker bees), there are other dates when exposure sites showed a higher DNA fragmentation than the control site (such as 27 July for worker bees). These contrasting results are difficult explain without considering that also in the control site there could be diffused stress-sources able to modify the physiological levels of the considered biomarkers. 

In the multi-stress site, we wanted to evaluate the cumulative effects of two different stress factors: the exposure to pesticides (the same as the chemical site) and the presence of an electromagnetic field generated by an electric transport line located above the experimental hives. Electromagnetic fields are known to cause different biological effects such as oxidative stress, genotoxic effects and immune system dysfunctions, all observed on different animal species [109]. The negative effects of electromagnetic radiation emitted by antennas, cell phones and high voltage power lines have been studied in humans [110,111,112,113] and in animals, including mice [114], bats [115], birds [116,117] and insects [118]. On bees, both electromagnetic fields generated by cell phones [109,119,120] and those generated by high voltage electricity transport lines [121] were studied. In this study, the cumulative effect of chemical and electromagnetic field exposure (multi-stress conditions) showed the worst general health condition, considering colony survival, pathology emergence and behavioural anomalies such as abnormal honey storage and excess of drone brood deposition. For the number of queen changes and the queen mortality in the underbasket, double the rate occurred in than in the control and even in the chemical-stress site. In particular, behavioural anomalies accorded to literature studies, as they revealed how exposure of bees to ELF frequencies leads to an increase in motor activity, with transient increase in hive temperature, hive weight loss, queen loss with abnormal royal cell production, reduced capped brood and poor winter survival [42]. In addition to electromagnetic field stress, the chronic exposure to some pesticides at sub-lethal concentrations is known to reduce the ability of overwintering and compromise the ability to feed and grow self-sufficient queens and drones [98]. In the present study, the combination of both factors in the multi-stress site contributed to the detrimental effects observed at this site, confirming the chronic effects of chemical and electromagnetic field exposure.

By analyzing the biomarker values in the multi-stress site, it can be observed that they generally follow those in the chemical site without a clear multi-stress effect. However, there are several specific effects observed only in the multi-stress site, especially at the end of the season (the last sampling on the 14th of October). The first one is the significantly lower increase, both in pupae and in worker bees, of the AChE activity in respect to the control and to the chemical-stress site. In literature, some studies carried out on mammals show that ELF in the interval between 50 and 90 Hz decreases the activity of this enzyme [122]. Another interesting result, observed in the last sampling (14 October), refers to the ALP over-activation both in worker bees and pupae with respect to the control and to the chemical-stress site (Figure 8). This result accords to a previous finding of [11] and suggests that this enzymatic over-activation at the end of the season can be related to the presence of an electromagnetic field at low frequencies, according to several studies carried out on different model organisms [43,123] including bees [42]. The observed enzymatic over-activation at the end of the season supports the hypothesis of a behavioural over-activation in specimens which, in contrast, should reduce their activity for preserving themselves for surviving the whole winter. The neurologic enzyme activity (AChE) and a concomitant metabolic over activation (ALP) may compromise specimen and colony survival during overwintering.

## 5. Conclusions

This research evidenced that both stress (chemical and electromagnetic) caused negative impacts on exposed colonies, due to disease appearance (American foulbrood), mortality in the underbaskets and behavioral alterations (queen changes, excess of drone brood deposition and honey storage). In detail, the mortality in the underbaskets appeared to be more related to the chemical-stress site while most of the behavioral alterations appeared only in the multi-stress one. Oveall, the loss of three out of four families in the multi-stress site confirmed the role of the multi-stress conditions as the mechanism able to cause the phenomena of hive depopulation (CCD).

Even if this study did not allow for testing of the synergic vs. additive effect of the multi-stress conditions, as single stress was only tested for pesticide-stress, we suggest that at least additive effects can be defined for our multi-stress conditions as several studies on ELF-EF effects have reported sub-lethal effects on bee colonies.

The increase and decrease of enzymatic biomarkers detected in exposure sites in comparison to control evidenced a complex picture of the experimental trial. However, as biomarker analyses gave no evidence of additive or synergic effects, thus rises the hypothesis that the contrasting effect of different pesticides and of the two stress sources may have reduced their diagnostic power. Pesticide-stress evidenced clear effects on several well-known biomarkers such as AChE and CAT and indirect evidence of ROS increase and damages (LPO) in the pesticide-stress site was observed (role of diagnostic tool). The observed less pronounced increase of the AChE activity at end of the season in both life stages confirmed previous results of ELF-EF effects, and can be considered an additional element which may threaten winter survival. Furthermore, the observed ALP increase at the end of the season in both life stages confirmed the hypothesis of a behavioral over-activation of bee activity in this period when bees should reduce their metabolism for preparing themselves to overwinter; this effect indicates an altered physiological condition, which, in accordance with the literature, may reduce overwintering success; this research seems to suggest that ALP increase in both pupae and adult bees at the end of the season can be considered a prognostic signal of a reduced winter survival.

All the results achieved in the present research confirmed biomarker analyses as a useful diagnostic and prognostic tool.

## Figures and Tables

**Table 1:** Health status parameters considered in the trial from April 2017 until April 2018.

Type	Parameter	Period	Methodology
Biological Observation	Hive Inspection	Weekly	Comb inspection and visual determination of the relative amount of storages, brood and empty space, according to [45]
	Comb Analysis	Weekly	Software image analysis of the central comb of each hive
	Queen Activity	Weekly	Visual inspection of the queen presence and deposition
	Bee mortality	Weekly	Count of dead bees of different stages and caste in the underbaskets, according to [46]
Parasites and pathogens	Varroa	Weekly	Count of the fallen mites on adhesive sticky boards positioned on the bottom of each hives
	Varroa	Six time	Count of the fallen mites after powdered sugar application
	Virus	Monthly	Real-Time–PCR [47]
	American foulbrood	Weekly Monthly	Visual inspection of the colonies Spore culture method, according to [48]
Biomarker	Acetylcholinesterase	Monthly	Spectrophotometric method, according to [49]
	Catalase	Monthly	Spectrophotometric method, according to [50]
	Glutathione S-transferase	Monthly	Spectrophotometric method, according to [51]
	Alkaline phosphatase	Monthly	Spectrophotometric method, according to [52]
	Reactive Oxygen Species	Monthly	Spectrophotometric method, according to [53]
	Lipid peroxidation	Monthly	Spectrophotometric method, according to [54]
	DNA fragmentation	Monthly	Spectrophotometric method, according to [55]

**Table 2:** List of commercial products (expressed as Kg a.i./hectare) in alphabetic order and their active ingredients used in 2017 in the orchard farm where exposure site was located.

Product Use		Active Ingredient	Culture						Total
		Crop Surface (ha)	Apple 1.5	Pear 1.0	Peach 5.0	Abricot 0.2	Plum 1.5	Cherry 0.15	
			kg a.i.	kg a.i.	kg a.i.	kg a.i.	kg a.i.	kg a.i.	kg a.i.
Actara^®^ 240 SC	insecticide	thiamethoxam a.i. 216 g/Kg	0.11						0.11
Affirm^®^	insecticide	emamectin benzoate a.i. 9.5 g/kg		0.01			0.01		0.02
Aliette^®^	fungicide	fosetyl-aluminium a.i. 800 g/kg		9.6					9.6
Alsystin^®^ SC	insecticide	triflumuron a.i.480.7 g/L	0.12		0.72				0.84
Caddy^®^	fungicide	cyproconazole a.i. 100 g/kg	0.01	0.04	0.05		0.01		0.11
Confidor^®^ 200 SL	insecticide	imidacloprid a.i. 200 g/L		0.15					0.15
Coragen^®^	insecticide	chlorantraniliprole a.i. 200 g/L			0.08				0.08
Crittam WG^®^	fungicide	ziram a.i. 760 g/kg			7.6	3.04	6.08	1.52	18.2
Crittox^®^	fungicide	mancozeb 750 g/kg	7.5						7.5
Decis^®^ Jet	insecticide	deltamethrin a.i. 15 g/L	0.01	0.02	0.05		0.02	0.01	0.11
Decision^®^	insecticide	deltamethrin a.i. 15 g/L				0.01	0.02	0.01	0.04
Delan^®^ 70 WG	fungicide	dithianon a.i. 700 g/kg	6.65	3.15					9.8
Difcor^®^	fungicide	difenoconazole a.i. 250 g/L			0.13				0.13
Efuzin 355 SC^®^	fungicide	dodine a.i. 355 g/L			2.84				2.84
Enovit metile^®^ FL	fungicide	thiophanate-methyl a.i.417 g/kg		0.83					0.83
Fixormon ^®^	plant regulator	NAA (1-naphthylacetic acid) a.i. 85 g/L	0.03						0.03
Indar^®^	fungicide	fenbuconazole a.i. 50 g/L	0.03		0.24		0.05	0.05	0.37
Intrepid^®^	insecticide	methoxyfenozide a.i. 240 g/L	0.24	0.12					0.36
Iperion^®^	fungicide	copper oxychloride a.i. 375 g/kg	11.25	8.81	11.25	3.38	7.13	3.19	45
Klartan^®^	insecticide	tau-fluvalinate a.i. 240 g/L	0.14					0.05	0.19
Kohinor ^®^	insecticide	imidacloprid a.i. 200 g/L	0.2						0.2
LaserTM	insecticide	spinosad a.i. 480 g/L	0.12		0.36		0.14		0.62
Nimrod^®^	fungicide	bupirimate a.i. 250 g/L			2.0				2.0
Oleoter^®^	insecticide	miner al oil 688 g/L	27.52	17.2	34.4	10.32	10.32		99.8
Ovipron^®^	insecticide	miner al oil 800 g/L		3.2					3.2
Polithiol^®^	insecticide	mineral oil 420 g/L	8.4						8.4
Prodigy ^®^	insecticide	methoxyfenozide a.i. 240 g/L	0.11	0.32					0.43
Reldan^®^	insecticide	chlor py rifos-methyl a.i. 255 g/L	0.23		1.35				1.58
Scala^®^	fungicide	pyrimethanil a.i. 400 g/L	1.1	0.9					2
Spada^®^	insecticide	phosmet a.i. 177 g/kg		0.71	0.53				1.24
Switch^®^	fungicide	cy prodinil a.i. 375 g/kg					0.28	0.24	0.52
		fludioxonil a.i. 250 g/kg					0.19	0.16	0.35
Tebusip combi^®^	fungicide	tebuconazole a.i. 45 g/L		0.54					0.54
		sulfur a.i. 700 g/kg		8.4					8.4
Teldor^®^	fungicide	fenhexamid a.i. 500 g/L		0.63			0.13		0.76
Tiovit ^®^	fungicide	sulfur a.i. 800 g/kg	25.6		14.8	0.8			41.2
Trebon^®^	insecticide	etofenprox a.i. 287.5 g/L	0.12	0.17	0.45			0.06	0.8
Zetor^®^	insecticide	abamectin a.i. 18 g/L		0.02					0.02
Fungicide treatment n°			13	11	17	6	9	11	67
lnsecticide treatment n°			13	11	10	1	5	4	44
Total treatment n°			**26**	**22**	**27**	**7**	**14**	**1** **5**	**1** **11**
Fungicide kg a.i.			52.1	32.9	38.9	7.22	13.9	5.2	150
Insecticide kg a.i.			37.3	21.9	37.9	10.3	10.5	0.13	118
Pesticide kg a.i.			**89.5**	**54.8**	**76.9**	**1** **7.6**	**24.4**	**5.29**	**268**

**Table 3:** GLM analyses of honey, pollen, brood and empty space as dependent variables and “hive” “date” and “treatment” as factors: D.F. = degrees of freedom; F = Fisher’s value; *p* = probability of the null hypothesis.

Dependent Variable	Factor	D.F.	F	*p*
Honey	Hive	3;219	3.50	0.015 *
	Date	24;219	1.65	0.033 *
	Treatment	2;219	19.6	<0.001 ***
Pollen	Hive	3;218	0.17	0.92
	Date	24;218	2.9	<0.001 ***
	Treatment	2;218	3.9	0.022 *
Brood	Hive	3;220	1.4	0.25
	Date	24;220	12.8	<0.001 ***
	Treatment	2;220	3.9	0.022 *
Empty space	Hive	3;213	7.3	<0.001 ***
	Date	24;213	9.6	<0.001 ***
	Treatment	2;213	2.2	0.12

* Significant (*p* < 0.05); *** highly significant (*p* < 0.001).

**Table 4:** Mean number ± standard deviation (minimum-maximum interval in brackets) of dead specimens in 10 days found in the underbasket in control, chemical- and multi-stress sites.

Treatment	Dead Animals/10 days						
	Worker Bees		Drones		Pupae		Queens
	Normal	Deformed	Normal	Deformed	Workers	Drones	
Control	165 ± 119	4.4 ± 8.7	11 ± 23	1.9 ± 4.1	0.17 ± 0.65	0.32 ± 1.2	0.14 ± 0.54
	(3.8–706)	(0–48.8)	(0–188)	(0–28)	(0–5)	(0–8.8)	(0–3.8)
Chemical-stress	228 ± 336	2.2 ± 6.9	11 ± 19	3.2 ± 13.6	0.11 ± 0.56	0.80 ± 2.4	0.21 ± 1.3
	(6.3–1769)	(0–62.5)	(0–160)	(0–134)	(0–5)	(0–17.5)	(0–12.5)
Multi-stress	232 ± 286	2.4 ± 5.1	13 ± 14	2.9 ± 4.9	0.12 ± 0.42	2.1 ± 0.72	0.35 ± 1.3
	(6.3–2069)	(0–28.8)	(0–49)	(0–23)	(0–2.5)	(0–5)	(0–8.8)

**Table 5:** Number of data (N), mean value, standard deviation (St dev), minimum, maximum and percentiles of AChE activity (U/mg protein), CAT activity (U/mg protein), GST activity (U/mg protein), ALP activity (U/mg protein), ROS level (AU/g f.w.), lipid peroxidation (nmol TBARS/g f.w.) and DNA fragmentation (μg DNA_fram_/g f.w.) in pupae and worker bees.

Biomarker	Stage	N	Mean	St dev	Min	Max	Percentiles		
							25th	50th	75th
AChE	pupae	115	0.053	0.018	0.020	0.11	0.040	0.049	0.064
	worker	120	0.15	0.076	0.050	0.36	0.090	0.12	0.19
CAT	pupae	115	16.3	6.1	2.7	34	13.1	16.7	19.4
	worker	131	22.4	8.4	6.2	45	16.3	22.7	28.5
GST	pupae	115	0.37	0.086	0.20	0.53	0.30	0.37	0.44
	worker	131	0.26	0.066	0.17	0.52	0.22	0.25	0.29
ALP	pupae	104	0.004	0.003	0	0.010	0.002	0.003	0.005
	worker	135	0.014	0.008	0	0.040	0.009	0.013	0.018
ROS	pupae	111	3.3 × 10^4^	2.6 × 10^4^	5.2 × 10^3^	1.6 × 10^5^	1.6 × 10^4^	2.5 × 10^4^	3.9 × 10^4^
	worker	121	3.5 × 10^5^	2.4 × 10^5^	2.9 × 10^4^	1.0 × 10^6^	1.5 × 10^5^	3.1 × 10^5^	4.8 × 10^5^
LPO	pupae	88	1.7	2.0	0	14.3	0.60	1.2	1.8
	worker	124	4.7	3.4	0.4	18.1	2.3	3.6	6.0
FRAM	pupae	113	82	64	4.8	468	46	68	91
	worker	122	135	87	8.5	422	68	114	180

**Table 6:** GLM of the analyzed biomarkers as dependent variables and “date” “treatment” and “hive” as factors, considering the interaction between date and treatment (date*treat): D.F. = degrees of freedom; F = Fisher’s value; *p* = probability.

Dependent Variable	Factor	Pupae			Worker Bees		
		D.F.	F	*p*	D.F.	F	*p*
AChE	Date	8;316	10	<0.001 ***	8;331	34	<0.001 ***
	Treatment	2;316	9.9	<0.001 ***	2;331	2.3	0.10
	Hive	3;316	3.7	0.013 *	3;331	1.5	0.21
	date*treat	15;316	4.7	<0.001 ***	15;331	6.9	<0.001 ***
CAT	Date	8;316	11	<0.001 ***	8;339	15	<0.001 ***
	Treatment	2;316	3.2	0.044 *	2;339	0.1	0.9
	Hive	3;316	0.28	0.84	3;339	1.9	0.13
	date*treat	15;316	2.2	0.005 **	15;339	2.3	0.003 **
GST	Date	8;316	7.3	<0.001 ***	8;339	94	<0.001 ***
	Treatment	2;316	3.4	0.036 *	2;339	5.8	0.003 **
	Hive	3;316	3.1	0.027 *	3;339	0.91	0.44
	date*treat	15;316	5.0	<0.001 ***	15;339	5.4	<0.001 ***
ALP	Date	8;278	25	<0.001 ***	8;343	15	<0.001 ***
	Treatment	2;278	2.5	0.084	2;343	1.0	0.36
	Hive	3;278	3.7	0.012 *	3;343	1.2	0.30
	date*treat	15;278	9.4	<0.001 ***	15;343	4.6	<0.001 ***
ROS	Date	8;311	14	<0.001 ***	8;316	3.9	<0.001 ***
	Treatment	2;311	6.6	0.001 **	2;316	7.5	0.001 **
	Hive	3;311	2.2	0.087	3;316	2.4	0.069
	date*treat	15;311	8.9	<0.001 ***	15;316	0.78	0.71
LPO	Date	8;245	5.2	<0.001 ***	8;314	7.3	<0.001 ***
	Treatment	2;245	0.2	0.79	2;314	2.1	0.13
	Hive	3;245	2.6	0.051	3;314	1.3	0.27
	date*treat	15;245	3.0	<0.001 ***	15;314	2.6	0.001 **
DNA_FRAGM_	Date	8;308	14	<0.001 ***	8;321	9.9	<0.001 ***
	Treatment	2;308	3.8	0.024 *	2;321	1.2	0.30
	Hive	3;308	0.67	0.57	3;321	0.76	0.52
	date*treat	15;308	2.0	0.015 *	15;321	3.8	<0.001 ***

* Significant (*p* < 0.05); ** very significant (*p* < 0.01); *** highly significant (*p* < 0.001).

## Supplementary Materials

The following are available online at https://www.mdpi.com/article/10.3390/insects12080716/s1, Figure S1: Experimental hives in the three experimental sites: control site (A), pesticide-stress site (B), multi-stress site (C), Figure S2: Image elaboration of a comb side by Image J software. (A) Preparation for area calculation; (B) example of definition of the worker brood area, Figure S4: Adhesive sticky board positioned under the grid on the drawer at the bottom of the hives for collecting naturally fallen *Varoa* mites, Figure S5: Treatment schedule of the active ingredients used during 2017 (kg a.i. ha^−1^) in the orchard farm where exposure site was located, Figure S6: Toxicity ratio (bee cm^−2^) of the active ingredients used during 2017 in the orchard farm where exposure site was located. Figure S7. Meteorological data are taken from “Rivolta d’Adda” and “Cavenago d’Adda” meteorological stations of the meteorological network of the Regional Environmental Protection Agengy. The two meteorological stations are located 3 km Est from the control site and 14 km South-Est from the exposure site, respectively. Table S1: Pesticide dose (kg a.i. ha^−1^ and in μg a.i. cm^−1^), oral and contact acute toxicity (μg a.i. bee^−1^) and toxicity ratio (bee cm^−1^) of the active ingredients used during 2017 in the orchard farm where exposure site was located.
