# Supplementary material for: Combined Effects of Pesticides and Electromagnetic-Fields on Honeybees: Multi-Stress Exposure
Source: Insects. 2021 Aug 10;12(8):716. doi: 10.3390/insects12080716 (PMC8396937; doi:10.3390/insects12080716)
Supplement: Supplementary file 1 [file insects-12-00716-s001.zip › insects-1275945-supplementary.pdf]

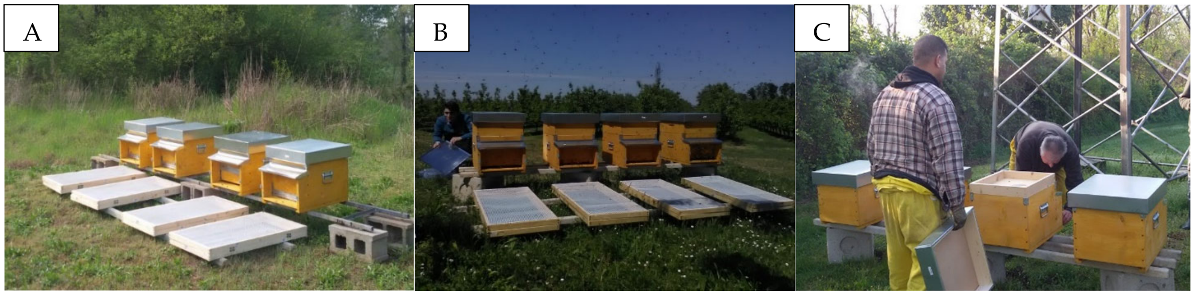

Figure S1. Experimental hives in the three experimental sites: control site (**A**); pesticide-stress site (**B**), multi-stress site (**C**).

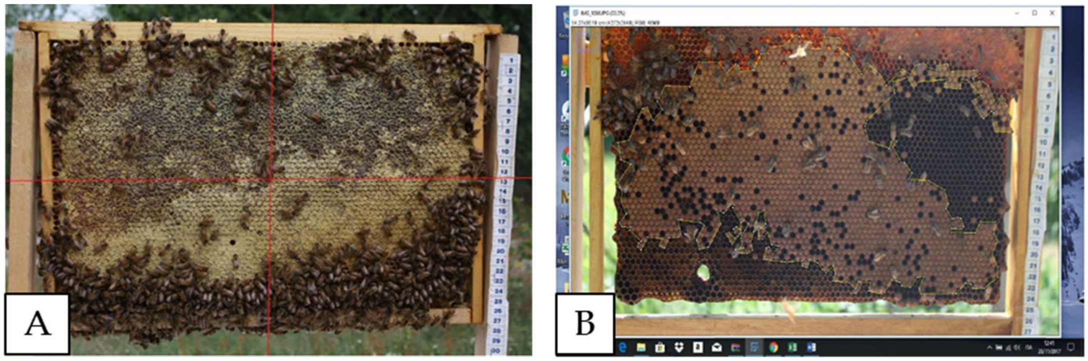

Figure S2. Image elaboration of a comb side by Image J software. (**A**) Preparation for area calculation; (**B**) example of definition of the worker brood area

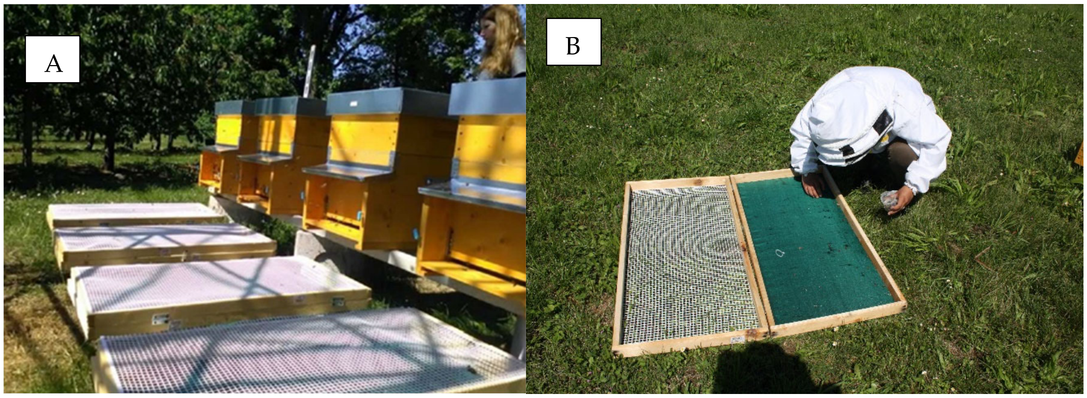

Figure S3. Underbasket positioned under the hives (**A**) and inspection (**B**)

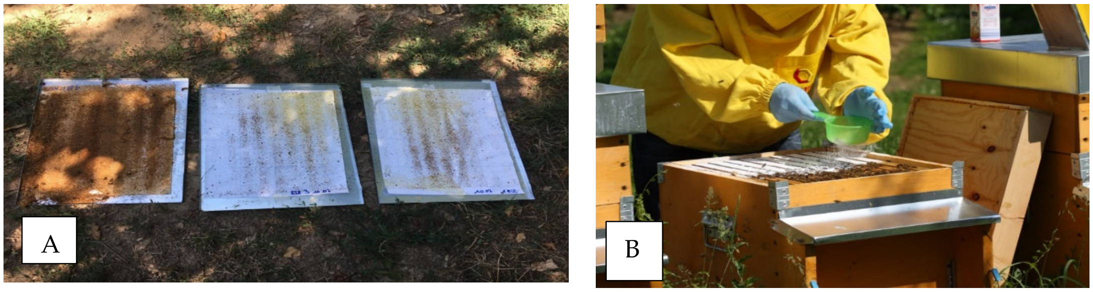

Figure S4. Adhesive sticky board positioned under the grid on the drawer at the bottom of the hives for collecting naturally fallen *Varoa* mites (**A**); Powdered sugar application for monitoring of *Varroa* mites (**B**).

2.2.4 Virus monitoring

Five bees were homogenized in 1 ml of MEM, which was clarified by a centrifugation lasting 5 minutes at 13000 rpm. Then, 200 µl of the supernatant were diluted in 800 µl di MEM for CBPV and ABPV, while for DWVs the whole supernatant was diluted in 4 ml of MEM and both were mixed by vortexing. Total RNAs were purified from 100 µl of clarified bee homogenate using NucleoMag Virus VET Kit (Macherey-Nagel) for ABPV/CBPV and One-For-All Vet Kit (Qiagen) with KingFisher Flex automated extraction system for DWV. The RNAs were recovered in 100 µl of elution buffer. Complementary DNA synthesis and amplification reaction were performed using QuantiTect Probe RT-PCR Kit (Qiagen) in a one-step RT-PCR Real-Time, with 20 µl of Mastemix and 5 µl of RNA according to the listed thermal cycling conditions: 30 min at 50°C (reverse transcription), 15 min at 95°C (initial denaturation) followed by 45 denaturation cycles at 95°C for 15s and annealing/extension at 60°C for 1 min for ABPV/CBPV virus, while for DWV the reverse transcription lasts only 20 min and the annealing/extension 70s.

The primers and probes used to quantify ABPV, CBPV and DWV are the following:

Virus	Forward primer 5′->3′	Reverse primer 5′->3′	Probe 5′→3′	Primer conc pmol/µl	Probe conc pmol/µl
ABPV	GCCCAGACAAGCGCAGTACT	AGCCGGAAAACGCGTCTT	(6-FAM)TCCCCGATAGCRACCGA (MGB)	50	5
CBPV	CGCAARTACGCCTTGATAAAGAAC	ACTACTAGAAACTCGTCGCTTCG	(6-FAM)TCAAGAACGAGACVACCGCCAAGTTC(BHQ-1)	50	5
DWV	ATGGGTTTGATTCG/AATATCTTGGAA	GATGTTCCG/AGGTGGCTTTAATGA	(6-FAM)ACTAGTGCTGGTTTTCCTTTGTC(MGB)	50	5

Moreover, five recombinant plasmids were produced in order to establish the standard curves of RT-qPCR Real Time at the following serial dilutions in TAE1X buffer: $2*10^6$, $2*10^5$,$2*10^4$,$2*10^3$, $2*10^2$ copies/µl. Threshold cycles from our samples were compared with standards curve establishing a linear relation. Results were expressed in viral genome copies per bee using the following equation: copies/bee= 200 x copies/PCR. This conversion factor was calculated based on the volume used at each step of the RT-qPCR Real Time method.

2.2.5 American and European foulbrood monitoring

For American foulbrood detection, direct inspection in field on combs and larvae was associated to analytical methods in laboratory where the identification of *P. larvae* was made from the debries and powdered sugar collected for *Varroa* monitoring.

On debris, the assays were performed as described by [1]: 1 g of debris was placed in a 15-mL test tube with a sealing cap containing 9 mL of sterile distilled water. After vigorous shaking by hand for 30 s, the suspension was heated in a water bath at 85 °C for 15 min to inactivate the thermosensitive contaminants. Immediately after the heat treatment, the suspension was poured into a stomacher bag with a lateral filter, and the filtered liquid was transferred with a disposable pipette into another test tube. The sample was plated onto five plates (100 µL/plate) of MYPGP (Mueller-Hinton broth, yeast extract, potassium phosphate, glucose and pyruvate) agar, supplemented with nalidixic acid and pipemidic acid).

On powdered sugar the following procedure was applied: one g of sugar was put in a 15 ml test tube with a screw cap containing 9 ml of sterile distilled water. The suspension was shaken by hand until the sugar was dissolved and then heated in a water bath at 85°C for 15 min. The homogenate was plated onto five plates (100 µl/plate) of MYPGP agar.

The culture plates were always incubated at 37 °C in an atmosphere with 10% CO_2 and examined after three and eight days. For each sample from two to five colonies with a *P. larvae*-like morphology were tested for catalase reaction and the catalase-negative colonies were subjected to Gram staining for confirmation.

When counting the colonies was not possible due to rapid growth of spore-forming bacteria or due to the presence of very large numbers of *P. larvae* colonies, ten-fold dilutions from the initial homogenate were prepared and then cultured with the previously described procedures. After numeration of *P. larvae* colonies, the number of viable spores was calculated and expressed as Colony Forming Units (CFU) per gram (debris or powdered sugar). The limit of detection (LOD) of the methods was 20 CFU/g.

2.2.6 Biomarker sampling and analyses

For the AChE assay, bees were homogenized in a sodium phosphate buffer (20 mM, pH 7.4), containing 250 mM sucrose and 1% Triton X-100, and processed as above. Enzymes were assayed spectrophotometrically. AChE was quantified at 412 nm in the presence of 0.5 mM acetylthiocholine iodide as substrate, as reported by [2].

CAT activity was determined according to [3] using H_2O_2 12 mM as substrate.

GST and ALP enzymatic activities were measured by homogenizing bee samples with nine volumes of ice cold Hepes–Tris 10 mM, pH 7.5, containing 50 mM mannitol and 1 mM dithiothreitol. The homogenate was filtered at 4 °C through surgical gauze to remove tissue debris. The crude extract was then centrifuged at 15,000 × g (4 °C) for 30 min to eliminate mitochondria. GST was assayed according to [4] through the measurement of glutathione-1-chloro-2, 4-dinitrobenzene conjugate production. ALP was assayed at 405 nm using p-nitrophenylphosphate as substrate.

All enzyme assays were performed in triplicate at 30 °C using sample volumes varying from 5 to 40 μl in 1 ml test cuvettes and a Cary3 UV–vis spectrophotometer. Enzyme activities were analyzed by Cary Win UV application software for Windows 2000, and expressed as international units (U) in μmol min^{-1} mL^{-1} and referred to protein concentration (mg ml^{-1}) as determined according to [5] using bovine serum albumin as standard.

For Reactive Oxygen Species (ROS), Lipid peroxidation (LPO) and DNA fragmentation (DNA_{fram}) analyses, adult bees were dissected in the head, thorax and abdomen. Only the head and abdomen were homogenized for biomarker analyzes. On the contrary pupae have been analyzed in full. Each sample was weighted and transferred to a new Eppendorf tube An homogenization potassium phosphate buffer was added at the ratio 1:10 (sample weight in mg: volume of homogenization buffer in μL; 100 mM potassium phosphate buffer with KCl 100 mM, EDTA 1 mM, protease inhibitors 1:100 v/v and dithiothreitol 1 mM, pH 7.4). Samples were homogenized within Eppendorf tube using an automatic tissue homogenizer. Two aliquots of 75 μL respectively of the homogenate were transferred to new Eppendorf tubes for the analysis of DNA_{fram} and 100 μL for the analysis of LPO, while the remaining homogenate was used for ROS measurement by dichlorofluorescein-diacetate (DCFH-DA) method, according to [6]. The remaining homogenate fraction was centrifugated at 15,000 x g at 4 ° C for 20 minutes, and the ROS analysis was carried out in duplicate directly in 96-well plates adding in each well 20 μL of supernatant, 100 μL of Phosphate-buffered saline (PBS), 8.3 μL of DCFH-DA 10 mg/mL in dimethyl sulfoxide (DMSO). For each multi-well plate, two blanks were prepared with the same reagents but substituting supernatant volume (sample) with the same volume of homogenization potassium phosphate buffer. Multi-well plate was incubated at 37 °C for 30 min. The fluorescence intensity was measured in duplicate by a TECAN Infinite spectrophotometer 200 PRO with excitation at λ 485 and emission at λ 536 nm, respectively. The ROS content was expressed as arbitrary units of fluorescence (AU) normalized to grams of extracted sample as fresh weight (AU g^{-1} f.w.). The average of the two blank readings was subtracted from the average of the two readings of each sample and the result was divided by the respective extracted weight in g.

Lipid peroxidation (LPO) was assayed spectrophotometrically by the determination of thiobarbituric acid-reactive substances (TBARS) according to [7]. In an Eppendorf, 100 μL of the homogenate (sample) was mixed to 500 μL of trichloroacetic acid (TCA) 12%, 400 μL Tris-HCl 0.5 M and 500 μL of thiobarbituric acid (TBA) 0.37%. Blanks were prepared with the same reagents but substituting homogenate volume (sample) with the same volume of homogenization potassium phosphate buffer. Samples and blanks were incubated at 90 °C for 1 h, refrigerated in ice for 10 min and centrifugated at 15,000 x g at 4 ° C for 10 minutes. 300 μL of the supernatant of samples and blanks were transferred to 96-well plates and the absorbance was read at 492 nm in duplicate by TECAN Infinite spectrophotometer 200 PRO. LPO was expressed as nmol TBARS formed per g of fresh weight (nMol g^{-1} f.w.), according to the Lambert-Beer's law:

$$\text{TBARS}\left(\frac{\text{nMol}}{\text{g f.w.}}\right) = \frac{(A_s - A_b) * \text{V}}{\varepsilon_{TBARS} * 10^{-9} * \text{l}} : sample\ weight\ (g\ f.w.)$$

with

A_s = mean absorbance of the two reading of each sample;

A_b = mean absorbance of the two blanks;

V = volume of the reaction (L): 1.5 * 10^{-3} L;

ε_{TBARS} = molar extinction coefficient for TBARS (1.56 *10^5 mol^{-1} * L * cm^{-1});

10^{-9} = equivalence factor for expressing TBARS in nMol;

l = optical path (cm): 1 cm.

DNA fragmentation was investigated by applying the DNA precipitation assay, according to [7]. In Eppendorf, 75 µL of homogenate was mixed with 600 µL of sodium dodecyl sulfate (SDS) 2%, EDTA 10 mM, Tris acetate 10 nM, NaOH 40 mM in ultrapure water. After one minute, 600 µL of KCl 0.12 M was added to induce precipitation of phosphates. Blanks were prepared in duplicate substituting homogenate volume (sample) with the same volume of homogenization potassium phosphate buffer. Samples and blanks were incubated at 60 ° C for 10 minutes under shaking, then they were cooled in ice for 30 minutes and centrifuged at 11,000 x rpm for 5 minutes. 375 µL of the supernatant obtained were then transferred in a new Eppendorf with 1125 µL of Hoechst (10 mg/mL). Samples were shaken for 5 minutes at 24 ° C and two aliquots of 300 µL were transferred in 96-well-plates and fluorescence was measured in duplicate by a TECAN Infinite spectrophotometer 200 PRO with excitation at ʎ 360 nm and emission at ʎ 465 nm. DNA was quantified by a calibration curve (3 - 100 µg/mL) obtained from genomic DNA extracted from salmon sperm. DNA fragmentation was expressed in µg of fragmented DNA per g of extracted fresh weight (µg DNA_{fram} g^{-1} f.w.), multiplying µg/mL by the sample volume (1.5 mL) and dividing by the extracted fresh weight (g).

Biomarker analyses were checked for repeatability both within-plate (intra-day variability) and among plates (inter-day variability); mean variation coefficients were 8.1% and 10.8%, respectively.

3. Results

3.1. Pesticide Exposure

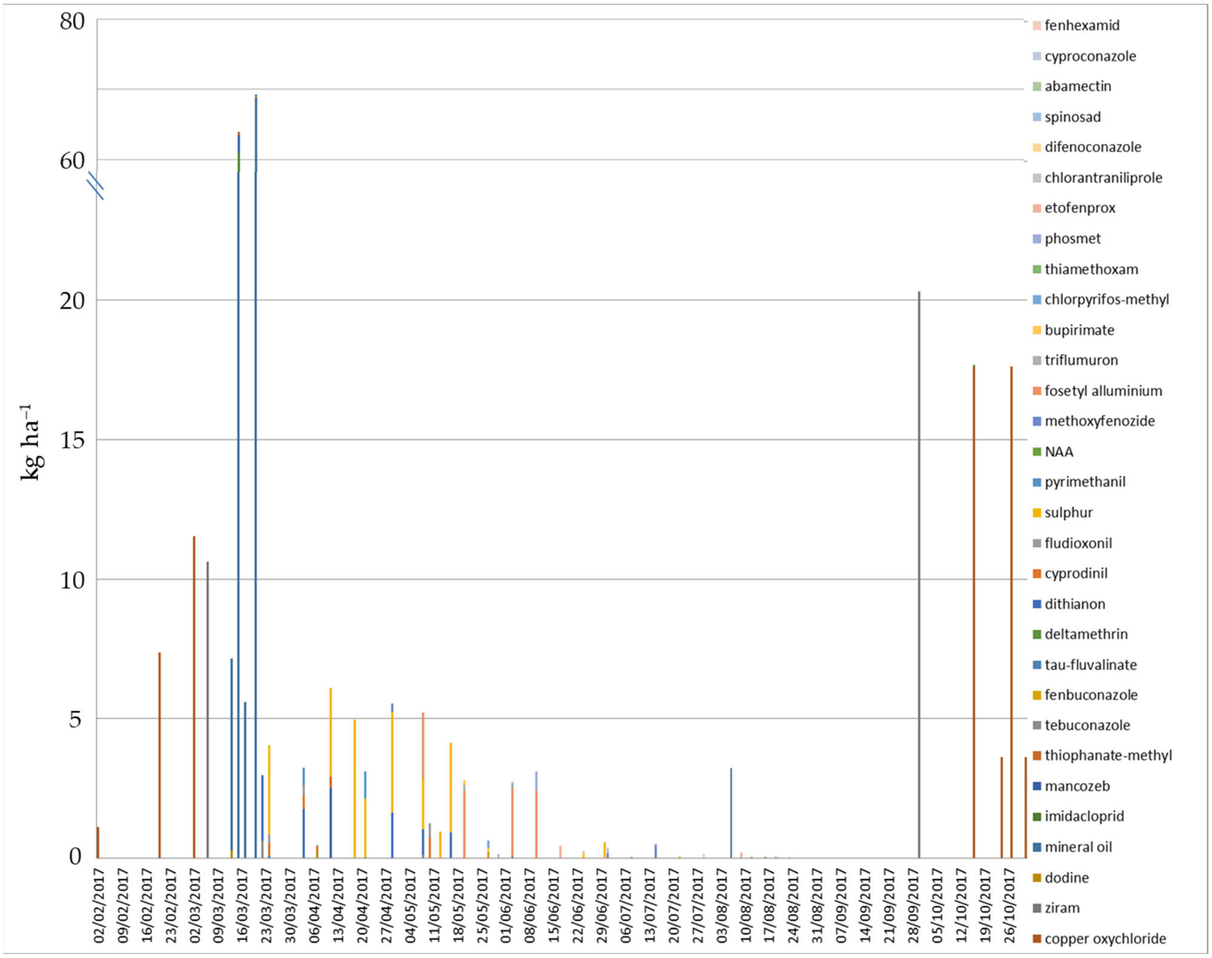

Figure S5. Treatment schedule of the active ingredients used during 2017 (kg a.i. ha⁻¹) in the orchard farm where exposure site was located.

Table S1 Pesticide dose (kg a.i. ha^{-1} and in μg a.i. cm^{-1}), oral and contact acute toxicity (μg a.i. bee^{-1}) and toxicity ratio (bee cm^{-1}) of the active ingredients used during 2017 in the orchard farm where exposure site was located.

Chemical Class	Active Ingredient	Use	Treatment Dose		LC_{50} Acute-48 h		Toxicity
			kg a.i. ha^{-1}	μg a.i. cm^{-2}	Contact μg Bee^{-1}	Oral μg Bee^{-1}	Ratio Bee cm^{-2}
Alkanes	mineral oil	insecticide	18.1	181	>3814	1474	0.12
Aminoglycoside	Spinosad	insecticide	0.041	0.41	0.05	0.049	8.3
Aminopyrimidine	pyrimethanil	fungicide	0.41	4.1	>100	>100	0.041
	bupirimate	fungicide	0.13	1.3	-	>200	0.0067
	cyprodinil	fungicide	0.36	3.6	>784	112.5	0.032
Anthranilic diamide	chlorantraniliprole	insecticide	0.020	0.20	>4	>104.1	0.050
Benzoylurea	triflumuron	insecticide	0.11	1.1	>200	>226	0.0056
Carbamate	Ziram	fungicide	5.2	52	>100	-	0.52
Diacylhydrazine	methoxyfenozide	insecticide	0.11	1.1	>100	>100	0.011
Dithiocarbamate	mancozeb	fungicide	2.5	25	193	193	0.13
Guanidine	Dodina	fungicide	0.28	2.8	>100	>200	0.028
Hydroxyanilide	fenhexamid	fungicide	0.069	0.69	>200	>102.07	0.0067
Inorganic compound	copper oxychloride	fungicide	3.5	35	-	12.1	2.9
	Sulfur	fungicide	2.0	20	>100	>106.8	0.19
Macrocyclic lactones	abamectin	insecticide	0.012	0.12	0.35	0.148	0.79
	emamectin benzoato	insecticide	0.012	0.12	0.0036	-	33
Neonicotinoid	imidacloprid	insecticide	0.061	0.61	0.081	0.0037	165
	thiamethoxam	insecticide	0.072	0.72	0.024	0.005	144
Organophosphate	chlorpyrifos-methyl	insecticide	0.21	2.1	0.059	0.25	36
	fosetyl-aluminium	fungicide	2.4	24	>1000	462	0.052
	Phosmet	insecticide	0.41	4.1	>1000	462	0.0088
Phenylpyrrole	fludioxonil	fungicide	0.081	0.81	>100	>100	0.0081
Pyrethroid	deltamethrin	insecticide	0.020	0.2	0.0015	0.074	133
	etofenprox	insecticide	0.12	1.2	>0.13	0.27	9.2
	tau fluvinate	insecticide	0.21	2.1	12	-	0.17
Quinine	dithianon	fungicide	0.95	9.5	>100	>25.4	0.37
Synthetic auxin	NAA	plant reg.	0.023	0.23	>120	-	0.0019
Thiourea	thiophanate-methyl	fungicide	0.42	4.2	>100	>100	0.042
Triazole	cyproconazole	fungicide	0.0079	0.079	>100	>100	0.00079
	difenoconazole	fungicide	0.025	0.25	>187	>187	0.0013
	fenbuconazole	fungicide	0.043	0.43	>5.5	>5.2	0.10
	tebuconazole	fungicide	0.23	2.3	>200	>83.05	0.028

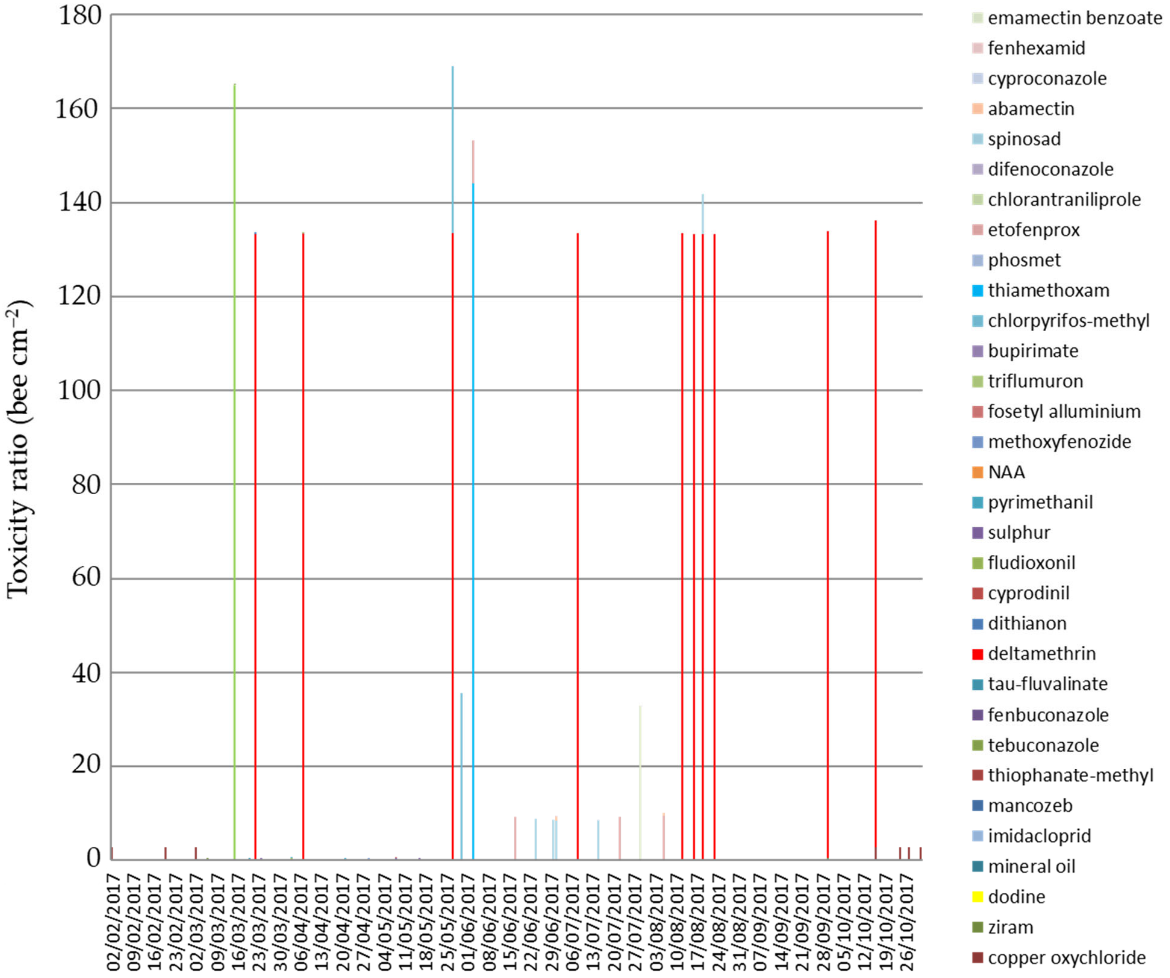

Figure S6 Toxicity ratio (bee cm^{-2}) of the active ingredients used during 2017 in the orchard farm where exposure site was located.

3.3 Meteorological conditions in the experimental sites

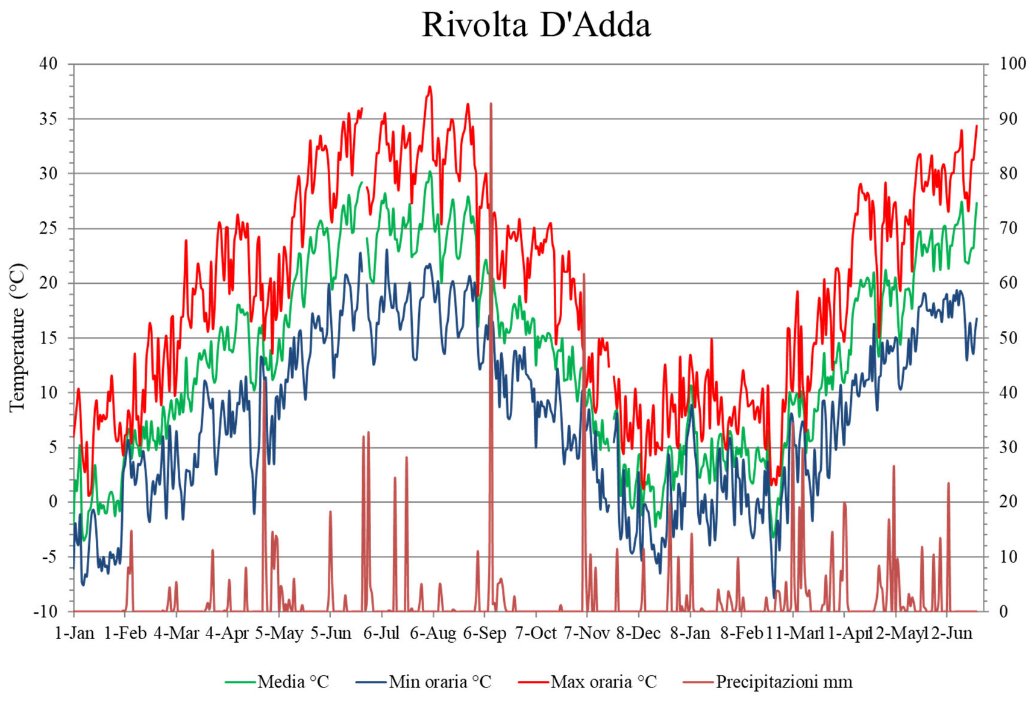

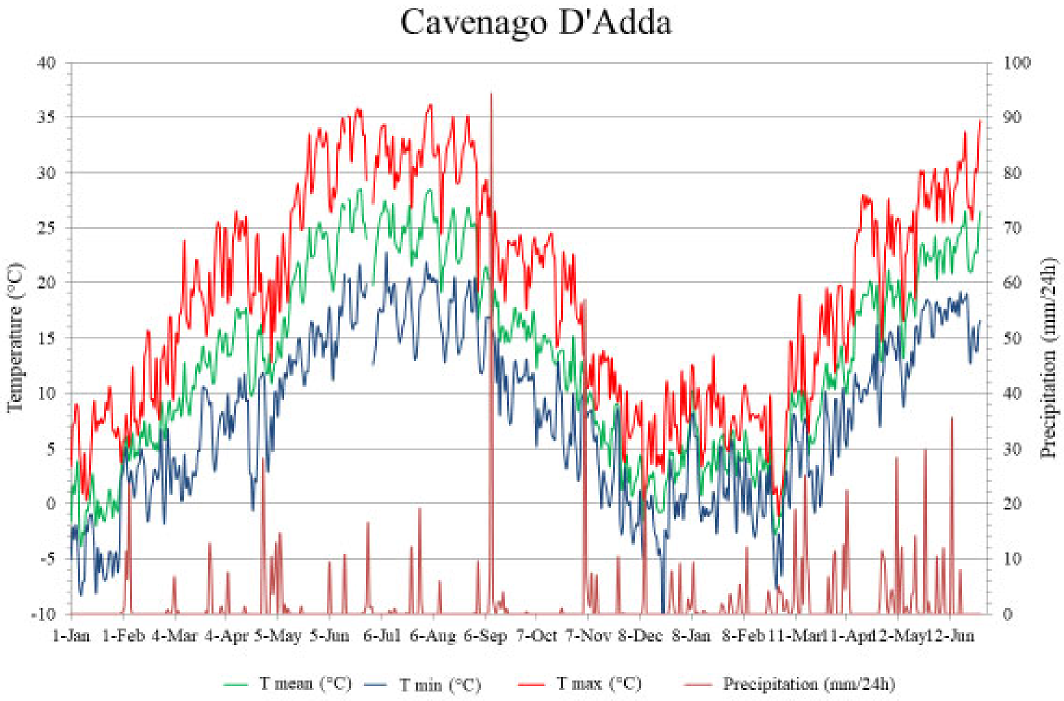

Figure S7. Meteorological data are taken from "Rivolta d'Adda" and "Cavenago d'Adda" meteorological stations of the meteorological network of the Regional Environmental Protection Agengy (https://www.arpalombardia.it/Pages/Meteorologia/Richiesta-dati-misurati.aspx). The two meteorological stations are located 3 km Est from the control site and 14 km South-Est from the exposure site, respectively.

References

1. Bassi, S.; Galletti, G. Detection of *Paenibacillus larvae* from beehive debris: A culture method based on watery extraction of spores. In Proceedings of the seventh European conference of apidology, Cluj Napoca, Romania, 7–9 September 2016.
2. Berra, E.; Forcella, M.; Giacchini, R.; Marziali, L.; Rossaro, B.; Parenti, P. Evaluation of enzyme biomarkers in freshwater invertebrates from Taro and Ticino river, Italy. *Int. J. Limnol.* **2004**, *40*, 169–180.
3. Bergmeyer, H.U. In *Methods of Enzymatic Analysis*; Bergmeyer, H.U., Ed.; Academic Press: Cambridge, MA, USA, 1983; Volume 2, pp. 165–166.
4. Habig, W.H.; Pabst, M.J.; Jakoby, W.B. Glutathione S-transferases. The fist enzymatic step in mercapturic acid formation. *J. Biol. Chem.* **1974**, *249*, 7130–7139.
5. Bradford M.M. A rapid and sensitive method for the quantitation of microgram quantities of protein utilizing the principle of protein-dye binding. Anal. Biochem. 1976, 72, 248–254.
6. Deng, J.; Yu, L.; Liu, C.; Yu, K.; Shi, X.; Yeung, L.W.; Lam, P.K.; Wu, R.S.; Zhou, B. Hexabromocyclododecane-induced developmental toxicity and apoptosis in zebrafish embryos. *Aquat. Toxicol*. **2009**, *93*, 29–36.
7. Olive, P.L. DNA precipitation assay: A rapid and simple method for detecting DNA damage in mammalian cells. Environ. *Mol. Mutagen*. **1988** *11*, 487–495.
